# The therapeutic effect of curcumin in metabolic dysfunction-associated steatotic liver disease: a systematic review and meta-analysis of animal studies

**DOI:** 10.3389/fphar.2025.1714245

**Published:** 2025-11-24

**Authors:** Xiao Li, Yiting Wang, Maocheng Xiong, Chunfang Xie, Dianxing Yang

**Affiliations:** 1 School of Basic Medical Sciences, Chengdu University of Traditional Chinese Medicine, Chengdu, China; 2 Department of Nuclear Medicine, Hospital of Chengdu University of Traditional Chinese Medicine, Chengdu, China

**Keywords:** curcumin, MASLD, liver-protective effect, meta-analysis, systematic review

## Abstract

**Background:**

The global prevalence of metabolic dysfunction-associated steatotic liver disease (MASLD) is rising sharply, driven by modern lifestyle and dietary changes. As MASLD threatens public health, exploring effective treatments is urgent. Curcumin may benefit MASLD by reducing inflammation and oxidative stress, but evidence reliability remains unclear. This systematic review and meta-analysis aims to evaluate curcumin’s effects in MASLD via animal studies.

**Methods:**

Relevant animal studies were searched in PubMed, Web of Science, Embase, China National Knowledge Infrastructure (CNKI), and Wanfang Database. Two researchers screened literature and extracted data; discrepancies were resolved via consultation. The SYstematic Review Center for Laboratory animal Experimentation (SYRCLE) risk of bias assessment tool was used to assess methodological quality. Meta-analysis followed the Cochrane Handbook, with analyses via RevMan 5.4 and STATA 15. The study was registered on PROSPERO (CRD42024553149).

**Results:**

A total of 22 studies were included, involving 430 animals. Compared with the control group, curcumin significantly reduced alanine aminotransferase (ALT), aspartate aminotransferase (AST), total cholesterol (TC), triglycerides (TG), low-density lipoprotein cholesterol (LDL) (alleviated dyslipidemia), NAFLD Activity Score (NAS), body weight, liver weight, liver index and inflammatory cytokines (tumor necrosis factor-α (TNF-α), interleukin-1 (IL-1), and interleukin-6 (IL-6)). It also increased high-density lipoprotein cholesterol (HDL). High heterogeneity was observed for ALT, AST, TC, TG, and HDL. Subgroup analyses showed HDL or LDL heterogeneity was likely associated with curcumin dose. For ALT, AST and LDL, duration might serve as a key regulatory factor contributing to their heterogeneity.

**Conclusion:**

Curcumin protected the liver from MASLD via anti-inflammatory, antioxidant, lipid metabolism-regulating, and insulin sensitivity-improving effects, thereby emerging as a therapeutic option for this condition. Limitations include low methodological quality of included studies and potential publication bias. Future research should use rigorous designs, large samples, and long-term studies to confirm efficacy/safety and clarify mechanisms.

## Introduction

Metabolic dysfunction-associated steatotic liver disease (MASLD), previously known as nonalcoholic fatty liver disease (NAFLD), is the prevalent chronic liver condition affecting approximately 25%–40% of the global population (Younossi et al., 2016; Fatima et al., 2024). It is characterized by the accumulation of triglycerides (TG) in liver cells, known as liver cells (Younossi et al., 2018; Wu et al., 2024). MASLD encompasses a spectrum of clinical and pathological manifestations, ranging from non-alcoholic fatty liver to metabolic dysfunction-associated steatohepatitis (MASH), which can further progress to severe conditions such as liver cirrhosis and hepatocellular carcinoma (HCC) (Abdul-Hai et al., 2015). MASLD is a growing global health concern, affecting approximately 32.4% of the world’s population (Zaiou and Joubert, 2025). Several factors contribute to the development and progression of MASLD, including obesity, dyslipidemia, insulin resistance, type 2 diabetes mellitus (DM2), and cardiovascular disease (Byrne et al., 2009). Currently, there are no approved pharmaceutical interventions specifically designed for the prevention or treatment of MASLD (Bhatia et al., 2012). Therefore, there is a critical need to explore and develop potentially effective therapeutic strategies to manage this complex liver disorder.

Natural products have been highly regarded for their potential in drug discovery (Li S. et al., 2023). Curcumin, a bioactive compound derived from turmeric, is renowned for its potent anti-inflammatory and anti-diabetic properties (Alrashdi et al., 2024). Curcumin exerted anti-inflammatory effects by inhibiting the nuclear factor κB (NF-κB) signaling pathway, thereby reducing the production of pro-inflammatory cytokines such as tumor necrosis factor-α (TNF-α), interleukin-6 (IL-6) (Zhai et al., 2024). In addition, curcumin activated the nuclear factor erythroid 2-related factor 2 (Nrf2) pathway, upregulate the expression of antioxidant enzymes heme oxygenase-1 (HO-1), NAD(P)H quinone oxidoreductase 1 (NQO1)), thereby alleviating oxidative stress-induced damage (Zhang et al., 2020). Oxidative stress was a core trigger for hepatocellular injury and the progression of steatosis (Machado et al., 2023). Research have indicated that supplementation with curcumin can ameliorate hepatic steatosis by modulating lipid metabolism in models. This regulation included the regulation of cholesterol, triglycerides (TG), and free fatty acid (FFA) in obese murine model (Hong et al., 2023; Yang J. et al., 2023; Fan et al., 2025). Furthermore, curcumin exhibited the ability to reduce plasma lipid levels and alleviated MASLD induced by a diet high in fats and fructose (Wang et al., 2016). Animal studies have shown favorable outcomes. These results highlight curcumin’s potential to manage dyslipidemia and MASLD (Ferguson et al., 2018). However, there are many different variables. These include dosage, how long the intervention lasts, sample sizes, and how curcumin is prepared. These variables mean we need to be careful. We should be cautious when interpreting curcumin’s protective effect on the liver. We also need to be careful when studying curcumin’s mechanisms. These mechanisms are for treating MASLD.

Furthermore, conducting a systematic review in the preclinical stage can clearly identify the areas that require further testing in animal experiments, prevent the occurrence of duplicate studies, and improve animal experiments. Hence, we undertook a systematic review and meta-analysis of preclinical animal research on curcumin’s efficacy on liver enzymes and metabolic factors in treating MASLD. The aim of this study is to demonstrate that curcumin has a protective effect on the liver in the treatment of MASLD. So we performed a systematic review and meta-analysis of preclinical animal studies to assess the effectiveness of curcumin.

### Literature search and review strategy

The meta-analysis and systematic review followed the Cochrane Handbook guidelines for Systematic Reviews of Interventions and adhered to the Preferred Reporting Items for Systematic Reviews and Meta-analyses. The meta-analysis protocol was registered with PROSPERO (CRD42024553149). Relevant animal studies published from inception to December 2024 were searched in electronic bibliographic databases such as PubMed, Web of Science, Embase, Wanfang Database and China National Knowledge Infrastructure (CNKI). The search was limited to Chinese and English languages. The medical subject terms (MeSH) and free terms used for database searches are “Non-alcoholic Fatty Liver Disease” or “Fatty Liver, Nonalcoholic” or “Fatty Livers, Nonalcoholic” or “Liver, Nonalcoholic Fatty” or “Livers, Nonalcoholic Fatty” or “Nonalcoholic Fatty Liver” or “Nonalcoholic Fatty Livers” or “NAFLD” or “Nonalcoholic Steatohepatitis” or “Nonalcoholic Steatohepatitides” or “Steatohepatitides, Nonalcoholic” or “Steatohepatitis, Nonalcoholic” or “MASLD” or “MASH” or “metabolic dysfunction-associated steatotic liver disease” or “metabolic dysfunction-associated steatohepatitis” and “Curcumin” or “Curcumin Phytosome” or “Phytosome, Curcumin” or “1,6-Heptadiene-3,5-dione, 1,7-bis(4-hydroxy-3-methoxyphenyl)- (E,E)-” or “Diferuloylmethane” or “Turmeric Yellow” or “Yellow, Turmeric” or “Mervia”. Additionally, Chinese databases were searched using the same Chinese search terms.

### Inclusion criteria

The inclusion criteria for including preclinical literature were as follows: 1) only animal models of simple steatosis or MASH are included, including subtypes with or without fibrosis (fibrosis stages F0-F4). 2) The animal type is limited to male rodents. Baseline weight and age requirements: mice weigh 18–25g, and rats weigh 120–240 g. Each group has a sample size of 6–40 animals, with consistent baseline characteristics between the intervention and control groups. 3) Only diet-induced models are included, including three specific types: high-fat diet (HFD) with a fat content ≥45%, fed continuously for ≥6 weeks. High-fat high-sugar diet (HSFD) containing ≥45% fat and ≥15% sugar (fructose or sucrose) of total calories, with a feeding duration of ≥10 weeks. Methionine-choline deficient diet (MCD) that lacks methionine and choline, administered for ≥6 weeks 4) The experimental group receives curcumin monotherapy (no combined medications) at all dosages. 5) The administration route is limited to gavage or intraperitoneal injection. 6) The intervention duration is 2–12 weeks 7) The model group receives an equivalent volume of vehicle (0.5% sodium carboxymethyl cellulose, dimethyl sulfoxide, carboxymethyl cellulose, phosphate-buffered saline), normal saline, or no treatment via the same route and for the same duration. 8) Primary outcome measures: Must include at least one of the following core outcome measures: alanine aminotransferase (ALT), aspartate aminotransferase (AST), total cholesterol (TC), triglycerides (TG), high-density lipoprotein cholesterol (HDL), low-density lipoprotein cholesterol (LDL), and NAFLD Activity Score (NAS). 9) Secondary outcome measures: May include animal body weight, liver weight, liver index, serum insulin, tumor necrosis factor-α (TNF-α), interleukin-1 (IL-1), and interleukin-6 (IL-6). 10) Language and publication type: Preclinical original research articles published in Chinese or English.

### Exclusion criteria

1) The types of study subjects not included are cirrhosis models, clinical trials, and *in vitro* studies. 2) Different Chinese herbal medicine. 3) Contrast with alternative substances. 4) Insufficient primary and secondary outcome data. 5) comment, conference, editorial, letter, reply. 6) Irrelevant or repetitive publishing. 7) Genetically modified models (ob/ob mice, db/db mice) and chemical toxin-induced models (induced by CCl_4_, D-galactosamine). 8) humans and other primate species.

### Study selection and data extraction

All the searched articles were imported into EndNote X9. Studies were selected and data were extracted in two phases. Initially, two reviewers screened the title and abstract of each study independently to identify those meeting the predefined inclusion criteria. Subsequently, the full text of potentially eligible studies was independently evaluated by the same two reviewers. Any disagreements about including a disputed study were resolved through discussion with a third reviewer.

Two researchers independently assessed and verified all included articles to refine data based on specific criteria: 1) recorded study ID with first author’s name and publication year. 2) extracted characteristics of various types including RCTs and experiments related to gender (male/female), sample size, model type, species, weight, etc. 3) detailed regimen features in intervention and comparison such as dosage, frequency, administration period, delivery route, vehicle. 4) outcomes in RCTs and experiments. 5) others. If a study had multiple experimental groups sharing a single control group, the control group was evenly divided among the comparisons, and each pair-wise comparison was included in the meta-analysis. In the same experiment, if there are multiple dose levels in the treatment group, the data from the highest dose group will be selected. if the study collects results at multiple time points, only the data from the last time point will be gathered for analysis. Any discrepancies in data extraction among reviewer authors were resolved by a third reviewer through discussion. All outcome measures are continuous data. Therefore, the mean and standard deviation corresponding to the intervention in each group should be recorded. If the study results are presented only in the form of charts, the researchers will attempt to contact the authors to obtain detailed data. If the authors cannot be reached to obtain the data, the GetData Graph Digitizer software will be used for digital extraction of the data from the charts.

### Risk of bias and quality of evidence assessment

The SYstematic Review Center for Laboratory animal Experimentation (SYRCLE) risk of bias assessment tool was used to assess methodological quality. The assessment includes ten items: (1) sequence generation. (2) baseline characteristics. (3) allocation concealment. (4) random housing. (5) blinding of experimenters. (6) random outcome assessment. (7) blinding of outcome assessors. (8) incomplete outcome data. (9) selective outcome reporting. (10) other sources of bias. Evaluation results were quantified, assigning each study a total score of ten, with one point per criterion. Two authors independently assessed study quality, resolving disagreements through discussion or consultation. In addition, the results of bias risk assessment for each section are categorized into three types: Yes (low risk of bias), No (high risk of bias), and Unclear (insufficient details reported to assess the risk of bias). If disagreements arise during the assessment process, they will be resolved through consultation with a third reviewer.

### Statistical analysis

This study adhered to the PRISMA guidelines for the design and reporting of statistical methods. The RevMan5.4 and STATA 15 software was utilized to analyze these data. Since the variable type data in this report is continuous, standardized mean differences (SMD) and 95% confidence intervals (CI) are used to represent the effect size. Statistical heterogeneity of our study was evaluated by using I^2^, the degree of heterogeneity was according the value of I^2^, and if the I^2^ ≤ or >50%. If I^2^ ≤ 50% indicated that there was low heterogeneity of our study, and a fixed-effect model was adopted to analyze the outcome data. If I^2^ >50% indicated that there was high heterogeneity of our study, and a random-effect model was adopted to analyze the outcome data. Due to significant heterogeneity, a random-effects model was used for the primary meta-analysis to pool the effect sizes. Given the presence of high heterogeneity, subgroup analyses were conducted for primary outcome measures to explore potential sources of heterogeneity. Subgroup stratification was based on five categories: curcumin dosage (divided into ≤100 mg/kg and >100 mg/kg according to the median dose of the included studies), modeling methods (high-fat diet [HFD], high-fat high-sugar diet [HSFD], and methionine-choline-deficient diet [MCD]), animal strains (mice and rats), and administration routes (intraperitoneal injection and gavage), and duration (divided into <8 weeks and ≥8 weeks according to the median dose of the included studies). This study conducted sensitivity analysis using a “leave-one-out” strategy to evaluate the stability of the results. Sensitivity analysis was performed when significant deviations were observed in the results of individual studies. For outcome measures with at least 10 included studies, potential publication bias was evaluated through two approaches: visual assessment via funnel plots and quantitative verification using Egger’s test. If publication bias was confirmed (asymmetric funnel plot and Egger’s test *P* ≤ 0.05), the Trim-and-fill method was applied to correct the pooled effect size. The structural diagram of curcumin was downloaded from the HERB (http://herb.ac.cn/) database.

## Results

### Research screening

**FIGURE 1:**
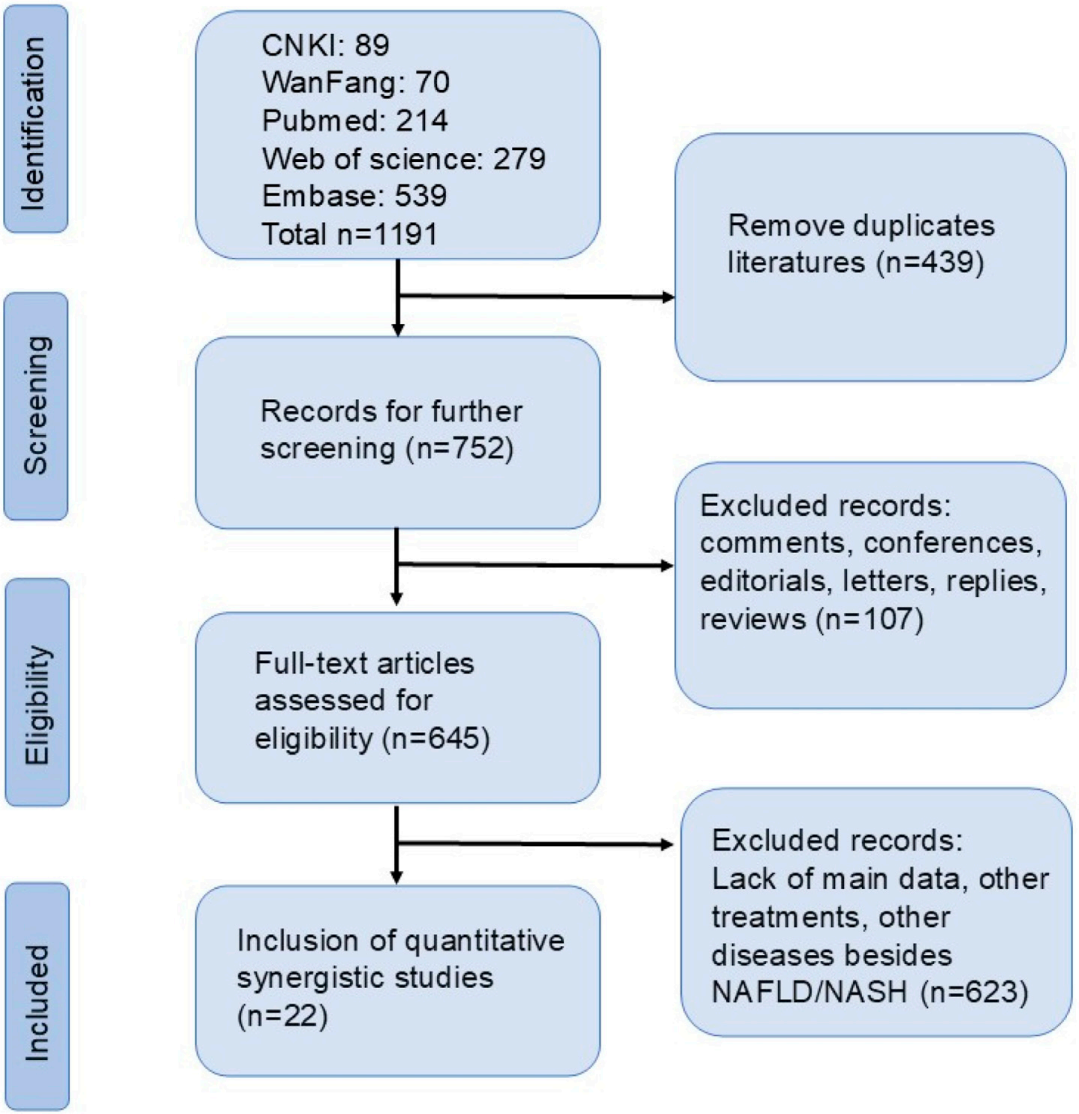
The flow chart of literature screening.

A total of 1,191 studies were found in the systematic review and meta-analysis database search. Among them, there were 89 from CNKI, 70 from Wanfang, 214 in PubMed, 539 in Embase, and 279 in Web of Science. After eliminating duplicates,752 publications were left. By reading the titles, abstracts, and full texts, finally, according to our exclusion criteria, eventually, 22 eligible studies were incorporated in the systematic review and meta-analysis. The study selection process was illustrated in Figure 1.

### Study characteristics

**FIGURE 2:**
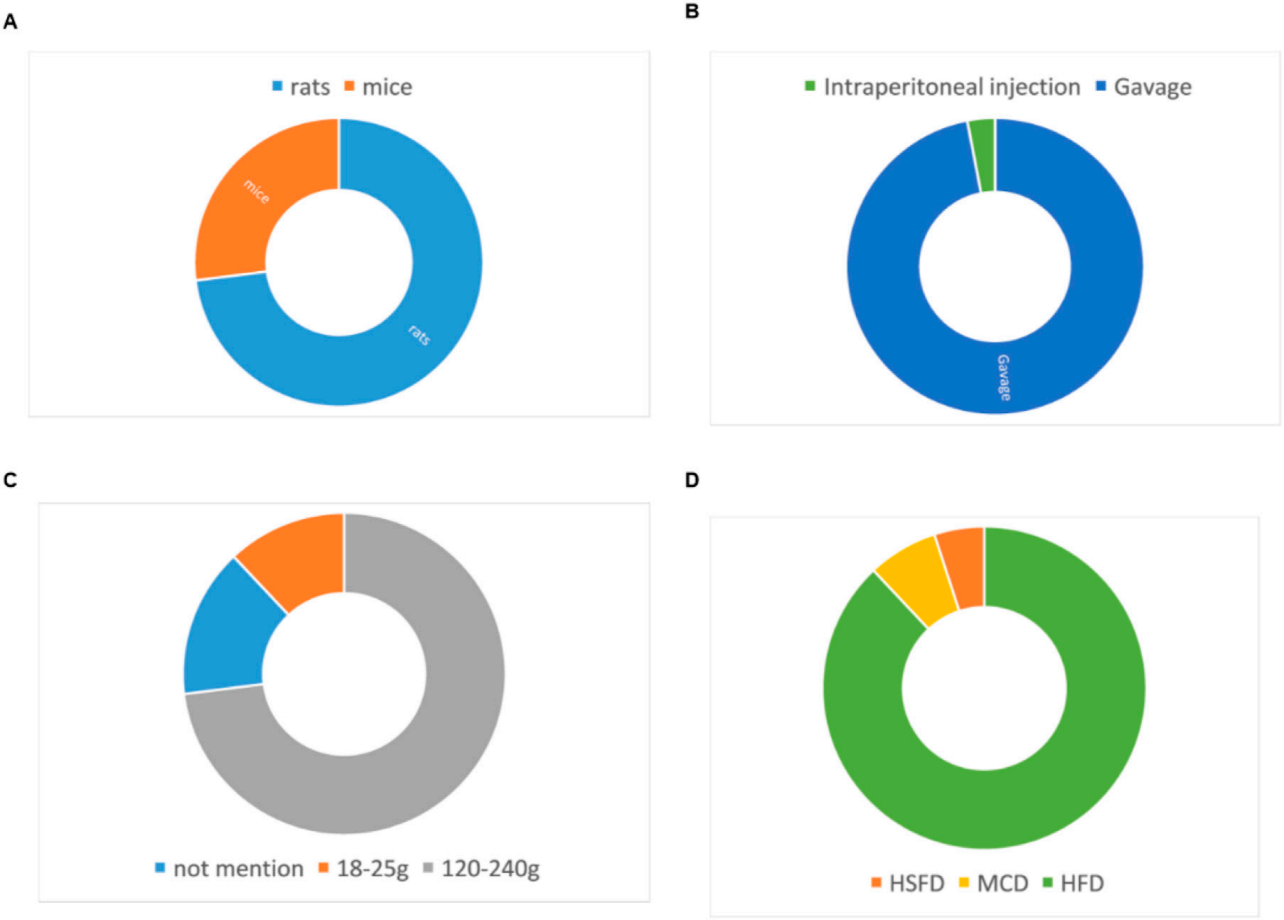
Study characteristics of studies. **(A)** animal strains, **(B)** administration route, **(C)** body weight, **(D)** modeling method.

22 eligible studies involving a total of 430 animals were selected. The experimental group comprised 200 animals, while the control group consisted of 230 animals. The animal species included mice and rats (Figure 2), seven studies of all which used mice (Chang et al., 2021; Vizzut et al., 2010; Afrin MR. et al., 2017; Yan et al., 2018; Tong et al., 2021; Zhang, 2022; Gu et al., 2024), two study used Male Wistar rat (Mai et al., 2011; Sun et al., 2021) and 13 studies used SD rats (Abd El-Hameed et al., 2021; Wu et al., 2023; Chen et al., 2015; Lin et al., 2024; Li, 2013; Hou et al., 2017a; Hou et al., 2017b; Gao, 2016; Xie et al., 2013; Shu et al., 2016; Xue et al., 2007; Wu et al., 2020; Tan et al., 2007). Rat weights varied between 120g and 240 g across all experiments, while mice weights ranged from 18 to 25 g. Four studies omitted animal weight data (Chang et al., 2021; Afrin MR. et al., 2017; Yan et al., 2018; Zhang, 2022). The studies featured three animal models: MCD, HSFD and HFD. Regarding the modeling methodologies employed, two studies conducted modeling using MCD (Vizzut et al., 2010; Tong et al., 2021), 19 studies adopted a HFD for the same purpose (Chang et al., 2021; Afrin MR. et al., 2017; Yan et al., 2018; Zhang, 2022; Gu et al., 2024; Mai et al., 2011; Sun et al., 2021; Abd El-Hameed et al., 2021; Wu et al., 2023; Chen et al., 2015; Lin et al., 2024; Li, 2013; Hou et al., 2017a; Hou et al., 2017b; Gao, 2016; Xie et al., 2013; Shu et al., 2016; Xue et al., 2007), and the remaining one study utilized a HSFD to establish their models (Tan et al., 2007). The curcumin dosages in these studies varied across multiple levels, ranging from 25 to 500 mg/kg/day. The maximum dose was 500 mg/kg and lasted for a total of 8 weeks. The detailed features were presented in Table 1.

**TABLE 1:** Characteristics of the included studies.

Study (year)	Species (sex, n = treatment/control group, age, and weight)	Model method	Curcumin group (administration, drug dose, duration)	Model group (administration, drug dose, duration)	Outcomes
Abd El-Hameed et al. (2021)	SD rats (male,10/10,150–200 g,6–8 weeks)	HFD	Gavage, 50 mg/kg, 2 weeks	No mention	AST, ALT, body weight, liver index, H&E
(Wu et al., 2023)	SD rats (male,6/6,160–180 g,6 weeks)	HFD	Gavage,25/50/100 mg/kg, 8 weeks	Normal water	AST, ALT, TC, TG, HDL, LDL, body weight, TNF-α, IL-1, H&E
Chang et al. (2021)	C57BL/6 mice (male, 10/10, 5 weeks)	HFD	Gavage, 100 mg/kg, 10 weeks	0.5% carboxy methyl cellulose	AST, ALT, TG, body weight, liver weight, serum insulin, H&E
(Afrin et al., 2017a)	C57BL/6 mice (male,6/6, 4 weeks)	HFD	Gavage, 100 mg/kg, 4 weeks	Normal water	TC, TG, body weight, H&E
(Yan et al., 2018)	C57BL/6 mice (male, 6/6, 4 weeks)	HFD	Gavage, 50/100 mg/kg, 4 weeks	0.5% sodium carboxymethyl cellulose	TC, TG, ALT, body weight, liver weight, liver index
Chen et al. (2015)	SD rats (male, 16/40,180–240 g)	HFD	Gavage, 50/100/200 mg/kg/d, 4 weeks	carboxymethyl cellulose	AST, ALT, TC, TG, H&E
Lin et al. (2024)	SD rats (male, 11/11, 200 g)	HFD	Gavage, 40 mg/kg, 4 weeks	Normal water	AST, ALT, TC, TG, LDL HDL, body weight, liver weight, liver index, H&E
(Zhang, 2022)	C57BL/6 mice (male, 10/10, 18–22 g,8–10 weeks)	HFD	Gavage, 150 mg/kg, 4 weeks	0.9% saline	ALT, AST, TG, IL-1β, IL-6, TNF-α, H&E
Mai et al. (2011)	Wistar rats (male, 10/10, 150–170 g)	HFD	Gavage, 50 mg/kg, 12 weeks	No mention	ALT, AST, TC, TG, H&E
Li (2013)	SD rats (male, 12/12, 190g–210 g)	HFD	Gavage, 50 mg/kg, 6 weeks	0.5% sodium carboxymethyl cellulose	ALT, AST, TG, TC, body weight, liver weight, liver index, TNF-α, IL-6, H&E
Hou et al. (2017a)	SD rats (male, 10/10, 175–225 g)	HFD	Gavage, 200 mg/kg, 8 weeks	Normal saline	AST, ALT, TNF-α, H&E
Hou et al. (2017b)	SD rats (male, 10/10, 175–225 g)	HFD	Gavage, 200 mg/kg, 8 weeks	Normal saline	AST, ALT, H&E
Sun et al. (2021)	Wistar rats (male, 8/8,180–220g, 8 weeks)	HFD	Gavage, 200/400 mg/kg, 8 weeks	Normal water	AST, ALT, TC, TG, HDL, TNF-α, serum insulin, H&E
Gu et al. (2024)	C57BL/6 mice (male, 8/8, 3–4 weeks)	HFD	Gavage, 200 mg/kg, 12 weeks	No mention	body weight, liver index, TC, TG, HDL, LDL, ALT, AST, H&E
Gao (2016)	SD rats (male, 8/8, 137.3–158.7g, 4 weeks)	HFD	Gavage, 200 mg/kg, 4 weeks	0.5% sodium carboxymethyl cellulose	liver weight, body weight, liver index, AST, ALT, TG, TNF-α, H&E
Xie et al. (2013)	SD rats (male, 6/6, 180–220 g)	HFD	Gavage, 200 mg/kg, 2 weeks	0.5% sodium carboxymethyl cellulose	AST, ALT, TC, TG, HDL, LDL, H&E
Shu et al. (2016)	SD rats (male, 9/9, 170–190 g)	HFD	Gavage, 400 mg/kg, 4 weeks	Normal saline	body weight, liver weight, TC, TG, ALT, AST, serum insulin, IL-6, TNF-α, H&E
Xue et al. (2007)	SD rats (male, 10/10, 170–240 g)	HFD	Gavage, 500 mg/kg, 4 weeks	Normal saline	Liver weight, ALT, TG, HDL, H&E
Wu et al. (2020)	SD rats (male, 10/10, 180–190 g)	HFD	Gavage, 500 mg/kg, 8 weeks	PBS	ALT, AST, TG, TC
c	C57BL/6 mice (male, 8/8, 18–20 g)	MCD	Gavage, 100 mg/kg, 8 weeks	0.5% sodium carboxymethyl cellulose	AST, ALT, TNF-α, IL-1β, H&E
Vizzut et al. (2010)	C57BL/6 mice (male,6/8,20–25g, 8 weeks)	MCD	Intraperitoneal injection,25 mg/kg/d,4/8/10 weeks	DMSO	ALT, body weight, liver weight, liver index, H&E
Tan et al. (2007)	SD rats (male, 10/14, 120–140 g)	HSFD	Gavage, 200 mg/kg, 12 weeks	Normal saline	AST, ALT, TC, TG, LDL, HDL, TNF-α, body weight, liver weight, liver index

### Study quality

**FIGURE 3:**
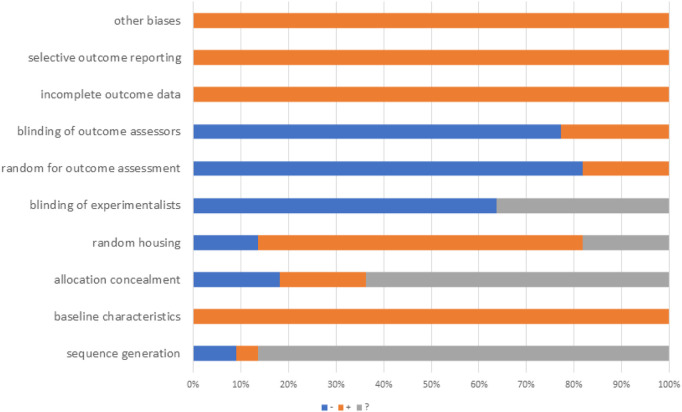
Study quality.

The SYRCLE risk of bias assessment tool was used to assess methodological quality. We conducted a rigorous quality assessment of the included studies, with detailed results presented in Table 2. The quality scores of all studies ranged from four to 5. Among the 22 included studies, 19 explicitly reported the use of random grouping methods. The baseline characteristics of animals were balanced across groups in all studies. However, several limitations in methodological reporting were identified. First, regarding allocation concealment, four studies provided no relevant information, while 14 studies had unclear reporting. Second, the blinding of experimenters was ambiguous in eight studies. Specifically, it remained unstated whether experimenters were blinded during the experimental process. Third, outcome assessment-related reporting was insufficient: 18 studies did not report randomness in outcome assessment, and 17 studies failed to report whether outcome assessors were blinded. Notably, no incomplete experimental data or selective reporting was observed across all studies. Additionally, no other potential sources of bias were identified in these included studies (Figure 3).

**TABLE 2:** Risk of bias of included studies.

ID	Sequence generation	Baseline characteristics	Allocation concealment	Random housing	Blinding of experimentalists	Random for outcome assessment	Blinding of outcome assessors	Incomplete outcome data	Selective outcome reporting	Other biases
Abd El-Hameed et al. (2021)	?	+	?	+	?	-	-	+	+	+
Wu et al. (2023)	?	+	?	+	?	-	-	+	+	+
Chang et al. (2021)	-	+	-	+	-	-	-	+	+	+
Vizzut et al. (2010)	?	+	?	+	-	+	+	+	+	+
Afrin et al. (2017a)	?	+	?	+	-	-	+	+	+	+
Yan et al. (2018)	?	+	?	+	?	-	-	+	+	+
Tong et al. (2021)	-	+	?	?	-	+	+	+	+	+
Chen et al. (2015)	?	+	?	+	-	-	-	+	+	+
Lin et al. (2024)	?	+	?	?	?	-	-	+	+	+
Zhang (2022)	?	+	+	+	-	-	-	+	+	+
Mai et al. (2011)	?	+	?	+	?	-	-	+	+	+
Li (2013)	+	+	?	+	-	-	-	+	+	+
Hou et al. (2017a)	?	+	?	+	-	+	+	+	+	+
Hou et al. (2017b)	?	+	?	+	?	+	+	+	+	+
Sun et al. (2021)	?	+	+	?	-	-	-	+	+	+
Gu et al. (2024)	?	+	+	+	-	-	-	+	+	+
Gao (2016)	?	+	?	+	-	-	-	+	+	+
Xie et al. (2013)	?	+	+	+	?	-	-	+	+	+
Shu et al. (2016)	?	+	?	?	-	-	-	+	+	+
Xue et al. (2007)	?	+	-	-	-	-	-	+	+	+
Wu et al. (2020)	?	+	-	-	?	-	-	+	+	+
Tan et al. (2007)	?	+	-	-	-	-	-	+	+	+

+, low risk of bias; −, high risk of bias; ?, unclear risk of bias.

### Primary outcome measure

#### Effect of curcumin on lipid content

##### Effect of curcumin on TG

**FIGURE 4:**
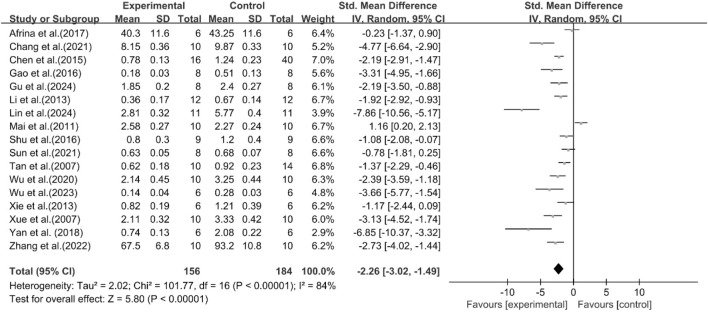
Forest plot: effect of curcumin on TG.

The excessive accumulation of TG in the liver is a pioneering event in the occurrence and progression of MASLD (Esler and Cohen, 2024). Among the included studies, 17 studies were conducted to assess the effect of curcumin on TG. The results demonstrated compared with the model group, curcumin group significantly decreased TG levels [n = 340, SMD = −2.26 [-3.02, −1.49]; heterogeneity: I^2^ = 84%, *P* < 0.05] (Figure 4).

##### Effect of curcumin on TC

**FIGURE 5:**
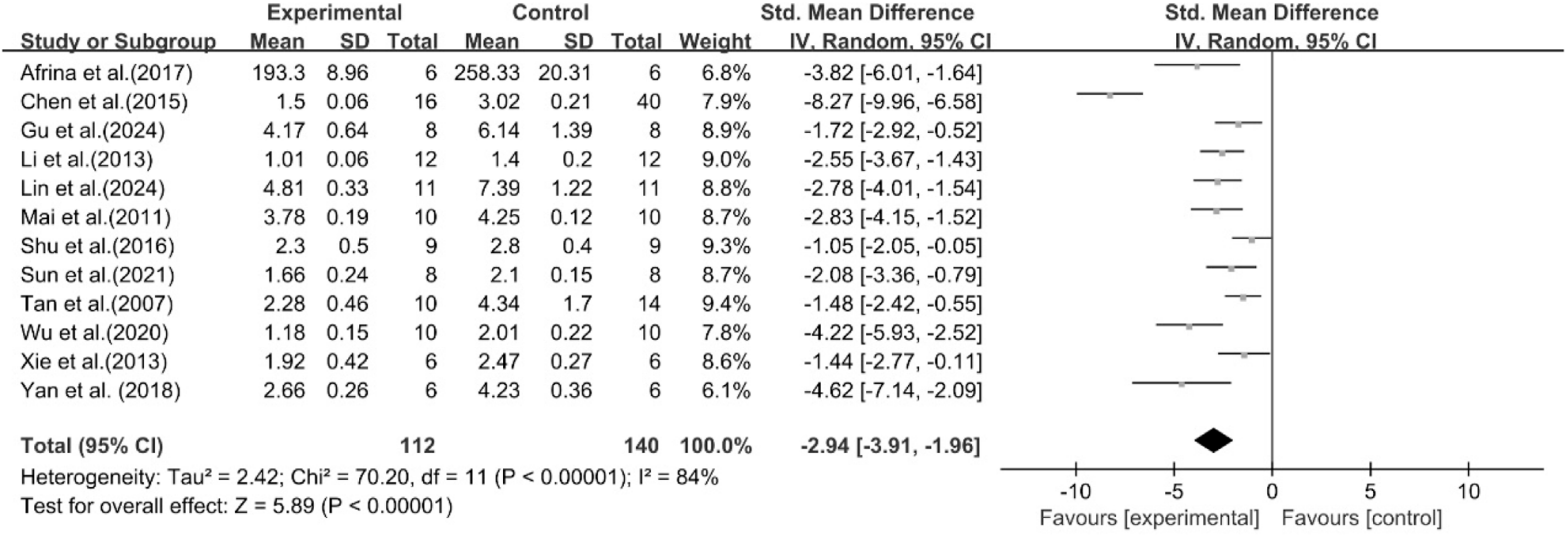
Forest plot: effect of curcumin on TC.

A total of 12 studies reported the effect of curcumin on TC levels. The results showed that compared with the model group, curcumin group significantly decreased TC levels [n = 252, SMD = −2.94 [-3.91, −1.96]; heterogeneity: I^2^ = 84%, *P* < 0.05] (Figure 5).

##### Effect of curcumin on HDL

**FIGURE 6:**
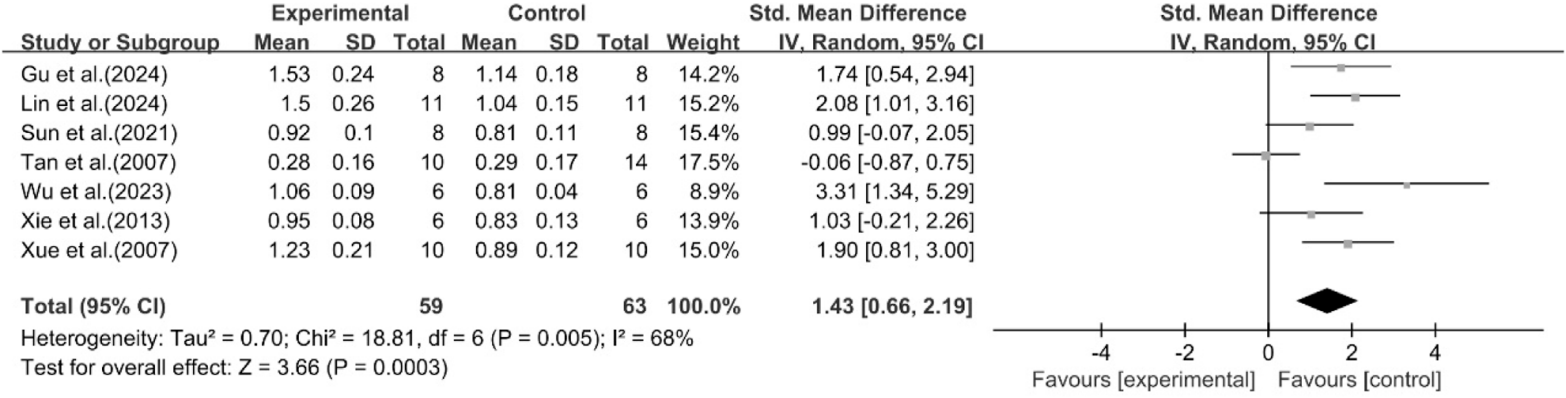
Forest plot: effect of curcumin on HDL.

A total of seven studies reported the effect of curcumin on HDL levels. The results showed that compared with the model group, curcumin group significantly increased HDL levels [n = 122, SMD = 1.43 [0.66, 2.19]; heterogeneity: I^2^ = 68%, *P* < 0.05] (Figure 6).

##### Effect of curcumin on LDL

**FIGURE 7:**
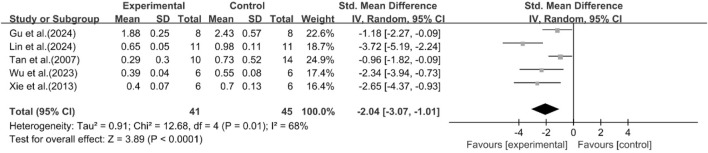
Forest plot: effect of curcumin on LDL.

A total of five studies reported the effect of curcumin on LDL levels. The results showed that compared with the model group, curcumin group significantly reduced LDL levels [n = 86, SMD = −2.04 [-3.07, −1.01]; heterogeneity: I^2^ = 68%, *P* < 0.05] (Figure 7).

### Histological scores

**FIGURE 8:**
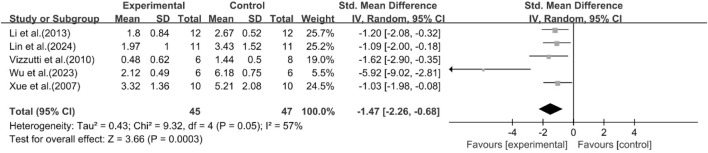
Forest plot: effect of curcumin on NAS score.

A total of five studies reported the effect of curcumin on the NAS score. The pooled analysis results showed that compared with the model group, the curcumin group significantly reduced the NAS score [n = 92, SMD = −1.47 [-2.26, −0.68]; heterogeneity: I^2^ = 57%, *P* < 0.05] (Figure 8). Due to the limited availability of existing research data, more studies on histology or inflammatory markers are needed to interpret and evaluate the above results more accurately.

### Effect of curcumin on liver enzymes

ALT and AST levels were defined as the primary outcomes to assess hepatocellular injury.

### Effect of curcumin on ALT

**FIGURE 9:**
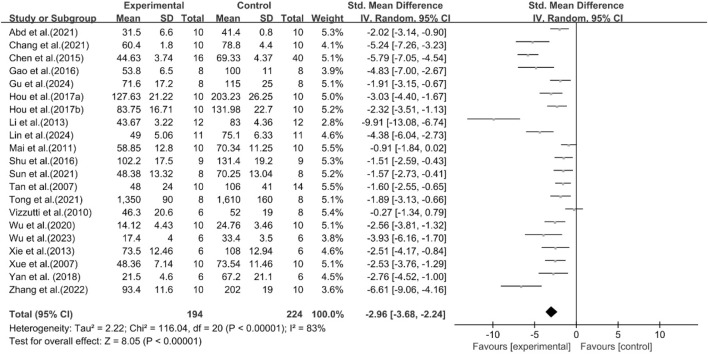
Forest plot: effect of curcumin on ALT.

A total of 21 studies reported the effect of curcumin on ALT. The pooled results showed that compared with the model group, curcumin group significantly reduced ALT levels [n = 418, SMD = −2.96 [-3.68, −2.24]; heterogeneity: I^2^ = 83%, *P* < 0.05] (Figure 9).

### Effect of curcumin on AST

**FIGURE 10:**
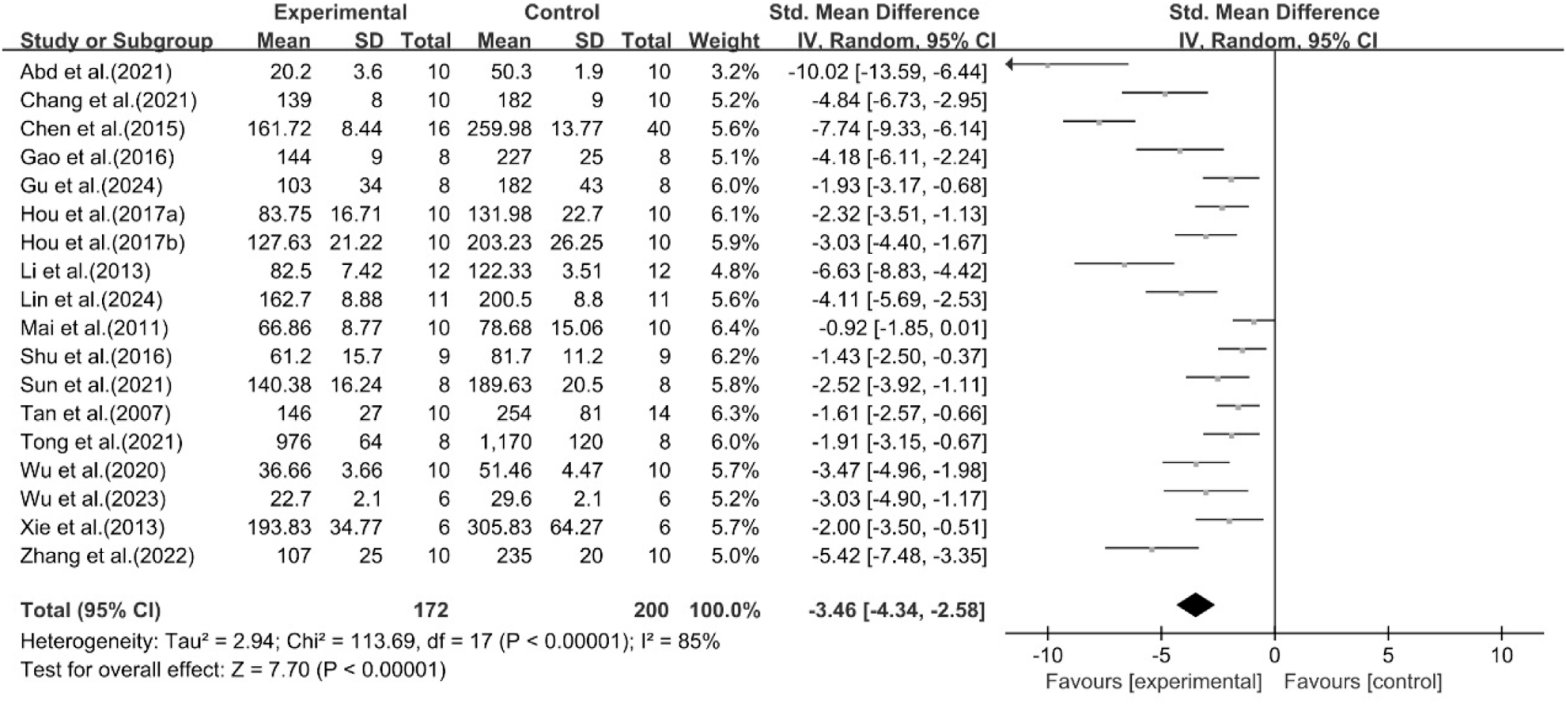
Forest plot: effect of curcumin on AST.

A total of 18 studies reported the effect of curcumin on AST. The pooled results showed that compared with the model group, curcumin group significantly reduced AST levels [n = 372, SMD = −3.46 [-4.34, −2.58]; heterogeneity: I^2^ = 85%, *P* < 0.05] (Figure 10).

**FIGURE 11:**
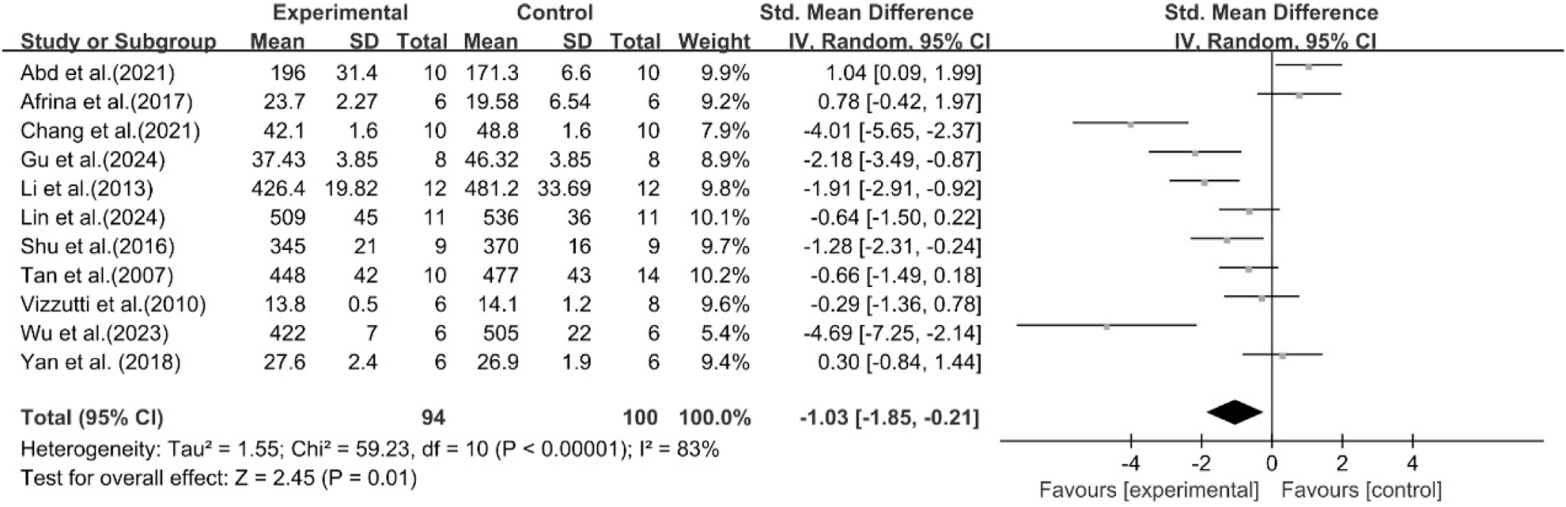
Forest plot: effect of curcumin on body weight.

A total of 11 studies reported the effect of curcumin on body weight. The pooled results showed that compared with the model group, curcumin group significantly reduced body weight [n = 194, SMD = −1.03 [-1.85, −0.21]; heterogeneity: I^2^ = 83%, *P* < 0.05] (Figure 11).

**FIGURE 12:**
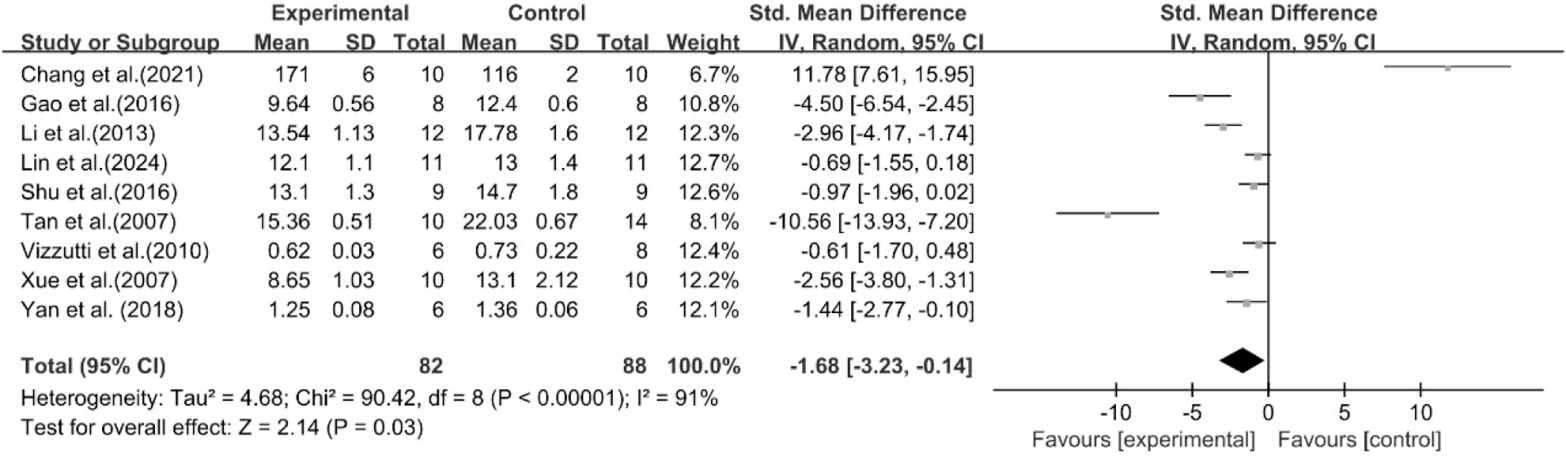
Forest plot: effect of curcumin on liver weight.

A total of nine studies reported the effect of curcumin on liver weight. The pooled results showed that compared with the model group, curcumin group significantly reduced liver weight [n = 170, SMD = −1.68 [-3.23, −0.14]; heterogeneity: I^2^ = 91%, *P* < 0.05] (Figure 12).

**FIGURE 13:**
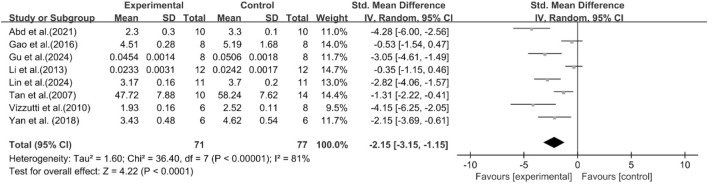
Forest plot: effect of curcumin on liver index.

A total of eight studies reported the effect of curcumin on liver index. The pooled results showed that compared with the model group, curcumin group significantly reduced liver index [n = 148, SMD = −2.15 [-3.15, −1.15]; heterogeneity: I^2^ = 81%, *P* < 0.05] (Figure 13).

**FIGURE 14:**
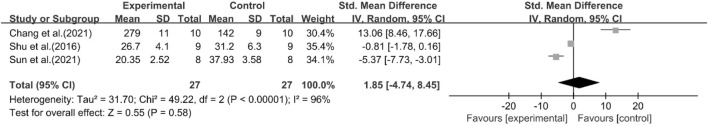
Forest plot: effect of curcumin on serum insulin.

A total of three studies reported the effect of curcumin on serum insulin. The pooled results showed that compared with the model group, curcumin group had no significant effect on serum insulin [n = 54, SMD = 1.85 [-4.74, 8.45]; heterogeneity: I^2^ = 96%, *P* > 0.05] (Figure 14).

### Effect of curcumin on inflammation-related indicators

#### Effect of curcumin on TNF-α

**FIGURE 15:**
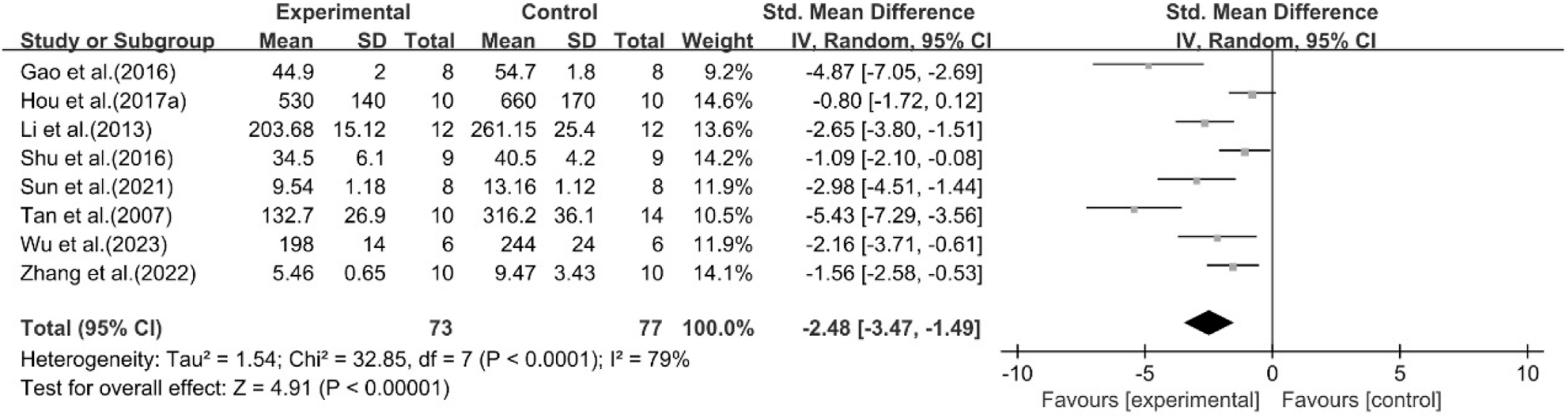
Forest plot: effect of curcumin on TNF-α

A total of eight studies reported the effect of curcumin on TNF-α. The pooled results showed that compared with the model group, curcumin group significantly reduced the expression level of TNF-α [n = 150, SMD = −2.48 [-3.47, −1.49]; heterogeneity: I^2^ = 79%, *P* < 0.05] (Figure 15).

#### Effect of curcumin on IL-1

**FIGURE 16:**
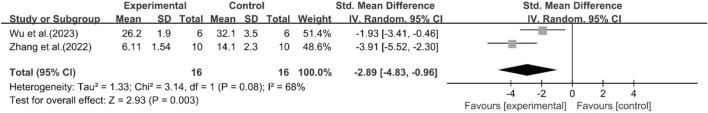
Forest plot: effect of curcumin on IL-1.

A total of two studies reported the effect of curcumin on IL-1. The pooled results showed that compared with the model group, curcumin group significantly reduced the expression level of IL-1 [n = 32, SMD = −2.89 [-4.83, −0.96]; heterogeneity: I^2^ = 68%, *P* < 0.05] (Figure 16).

#### Effect of curcumin on IL-6

**FIGURE 17:**
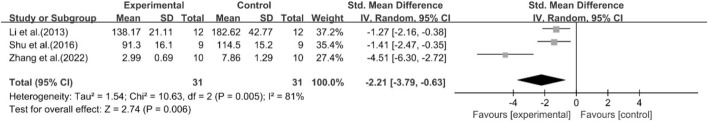
Forest plot: effect of curcumin on IL-6.

A total of five studies reported the effect of curcumin on IL-6. The pooled results showed that compared with the model group, curcumin group significantly reduced the expression level of IL-6 [n = 62, SMD = −2.21 [-3.79, −0.63]; heterogeneity: I^2^ = 81%, *P* < 0.05] (Figure 17).

### Subgroup analysis and sensitivity analysis

For outcome indicators with an I^2^ > 50%, a random-effects model was used for analysis in this study. Due to the high heterogeneity, we also conducted subgroup analyses of the main outcome indicators by animal strains, modeling methods, administration routes, and curcumin dosages. Subgroup analyses showed HDL or LDL heterogeneity was likely associated with curcumin dose. For ALT, AST and LDL, duration might serve as a key regulatory factor contributing to their heterogeneity (Supplementary Figures S1-S30; Supplementary Table S1). Additionally, even after subgroup analysis, some subgroups still exhibited high heterogeneity, which might be related to unidentifiable factors. Therefore, the pooled effect size of these subgroups should be considered for reference only.

**FIGURE 18:**
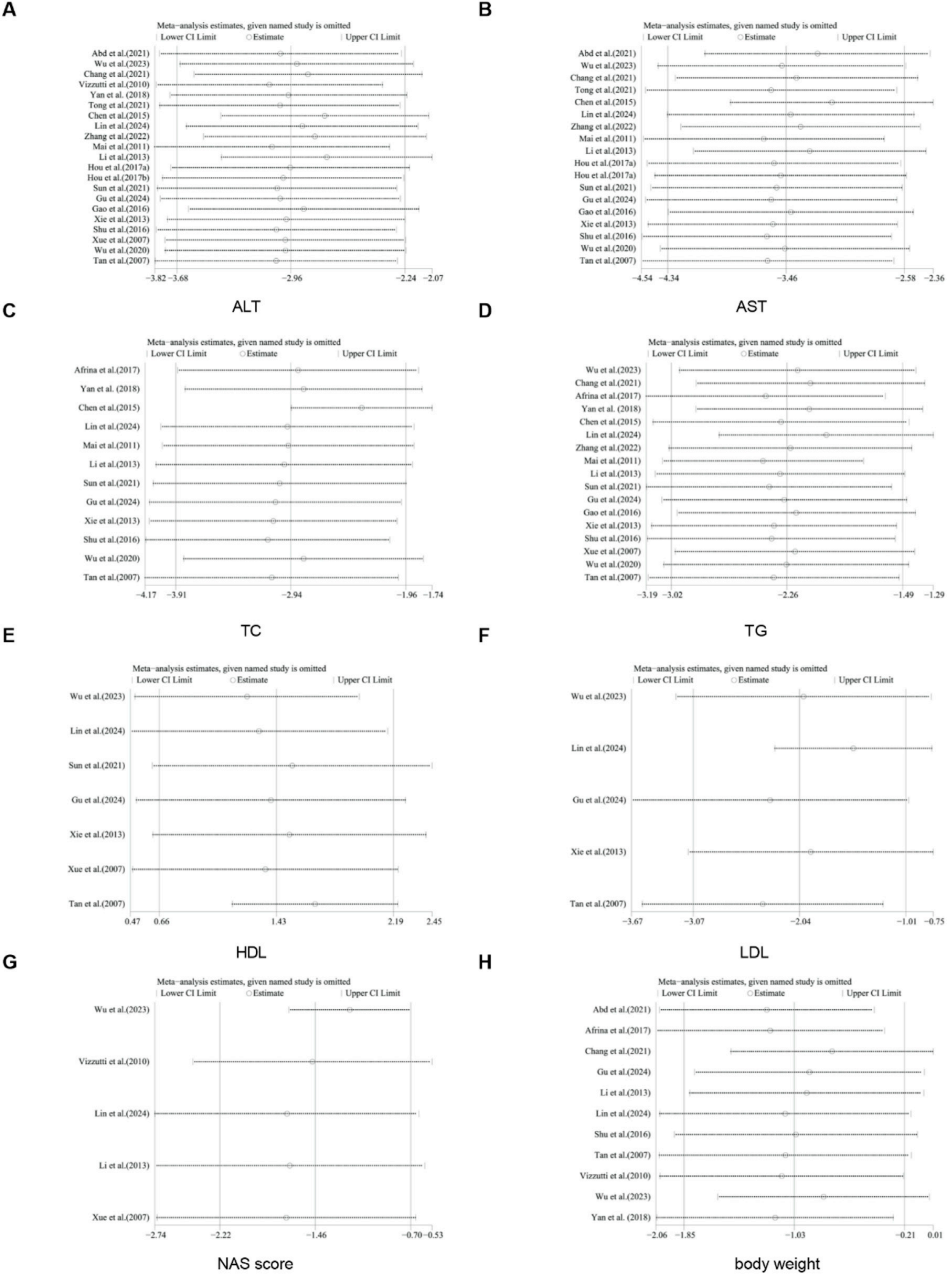
Results of sensitivity analysis. **(A)** ALT, **(B)** AST, **(C)** TC, **(D)** TG, **(E)** HDL, **(F)** LDL, **(G)** NAS score, **(H)** body weight.

**FIGURE 19:**
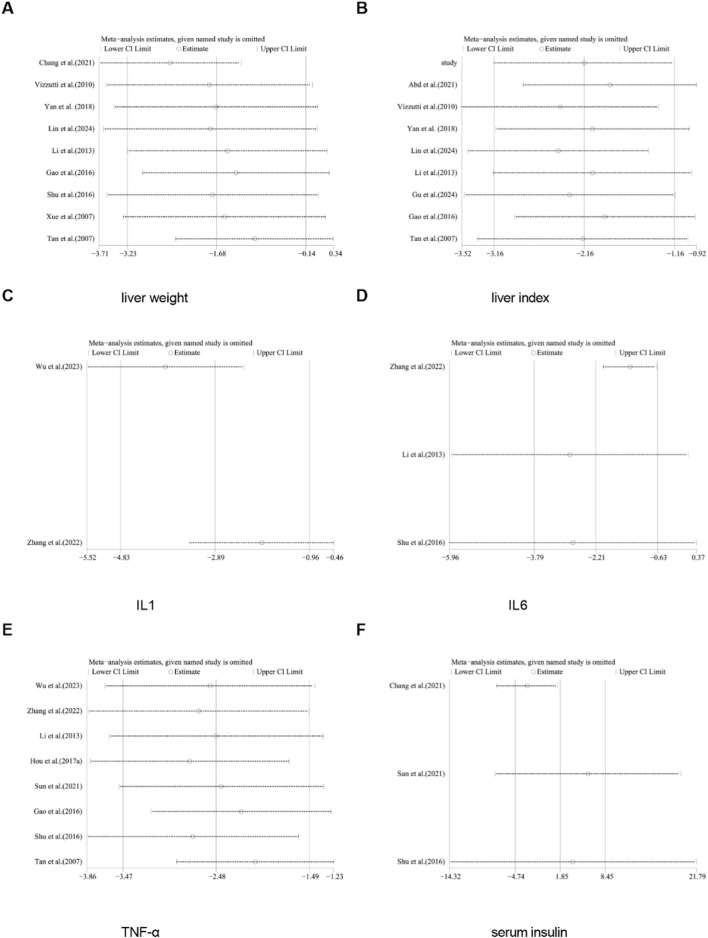
Results of sensitivity analysis. **(A)** liver weight, **(B)** liver index, **(C)** IL-1, **(D)** IL6, **(E)** TNF-α, **(F)** serum insulin.

For TC, TG, HDL, LDL, NAS score, ALT, AST, body weight, liver weight, liver index, serum insulin, TNF-α, IL-1, and IL-6, we conducted sensitivity analysis by removing one study at a time. The results indicated that removing any single study did not significantly affect the total effect size (Figures 18, 19).

### Publication bias

**FIGURE 20:**
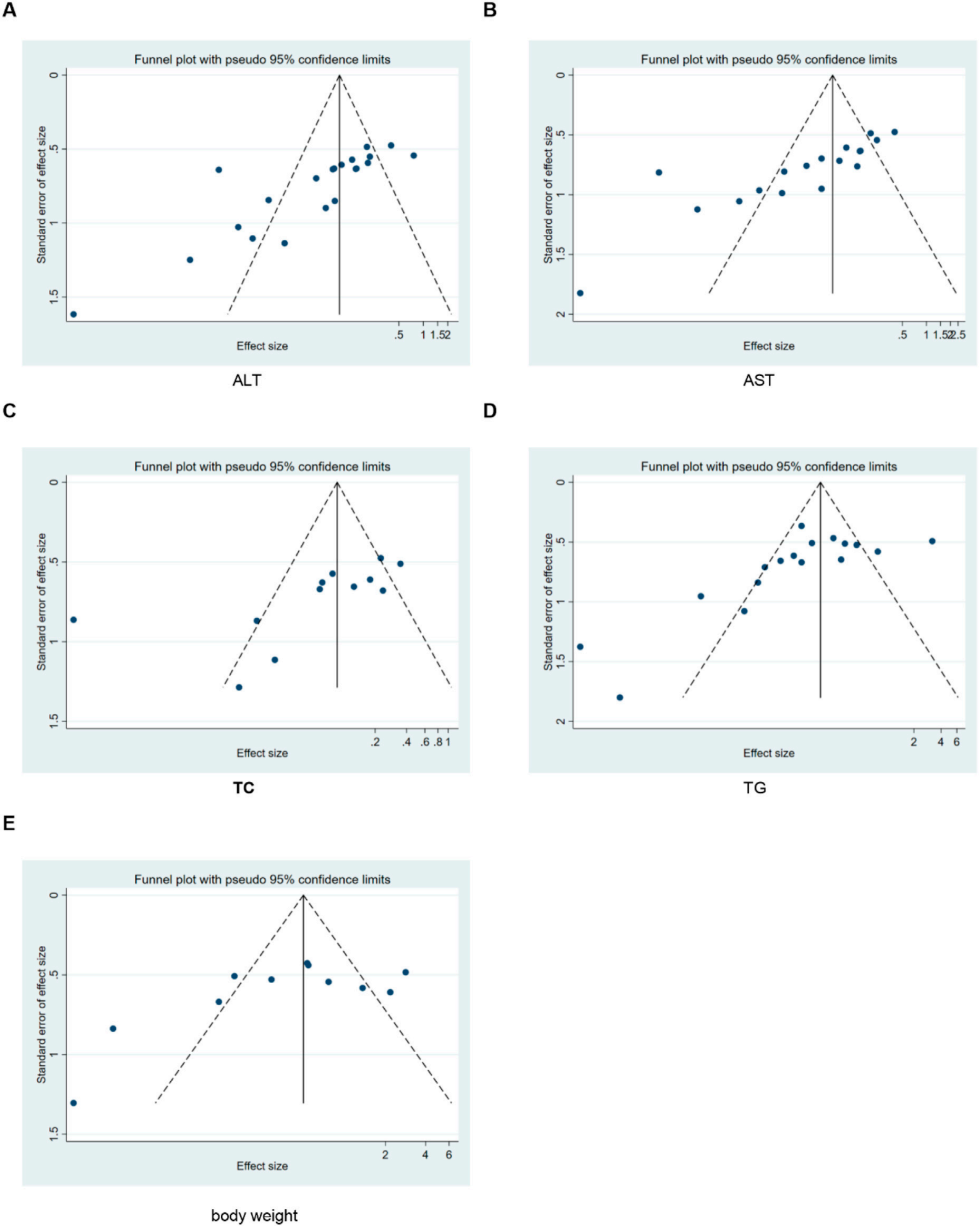
Results of funnel plots. **(A)** ALT, **(B)** AST, **(C)** TC, **(D)** TG, **(E)** body weight.

To further investigate publication bias, for each outcome indicator with no fewer than 10 included studies, we employed funnel plots and Egger’s test to assess potential systematic biases in the publication of research findings. The results revealed the presence of publication bias in some relevant indicators (Figure 20).

**FIGURE 21:**
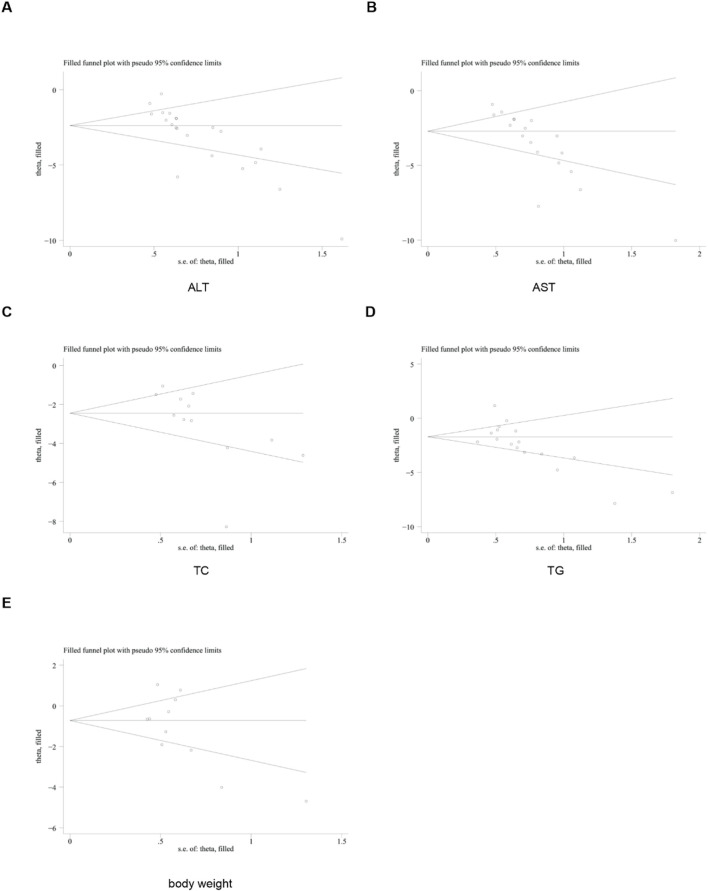
Results of trim and fill method. **(A)** ALT, **(B)** AST, **(C)** TC, **(D)** TG, **(E)** body weight.

Subsequently, the trim-and-fill method was utilized to evaluate the impact of this publication bias on the overall results. The findings of the trim-and-fill analysis indicated that these missing study data did not affect the stability of the results (Figure 21).

## Discussion

MASLD covers a spectrum from simple hepatic steatosis to MASH, cirrhosis, and even HCC (Li et al., 2025). It has become a major global public health concern (Yang et al., 2024). Effective clinical interventions for MASLD remain limited (Newsome et al., 2021). Thus, natural bioactive compounds like curcumin have drawn widespread attention. Curcumin is the primary active component of *Curcuma longa* (turmeric), a perennial herb (Stati et al., 2021). It is well-known for its anti-inflammatory, antioxidant, and metabolic regulatory properties (Askarizadeh et al., 2025). Before this research, no comprehensive meta-analysis had systematically quantified curcumin’s efficacy in preclinical MASLD animal models. To address this gap, we conducted a systematic review and meta-analysis. We performed a rigorous search across five major databases: CNKI, Wanfang, PubMed, Embase, and Web of Science. This search initially yielded 1,191 records. After removing duplicates, we screened titles, abstracts, and full texts against predefined criteria. Finally, 22 eligible studies were included, involving 430 animals. Among these animals, 200 were in the curcumin intervention group and 230 in the control group. These included studies had diverse designs. They covered three common MASLD modeling methods, multiple animal strains, and a wide range of curcumin dosages. We focused on three core therapeutic targets for MASLD: lipid metabolism regulation, liver function protection, and inflammation/oxidative stress inhibition. The pooled results provided potential reference of curcumin’s multifaceted beneficial effects in MASLD animal models.

Hepatic steatosis is the pathological hallmark of early MASLD (Egresi et al., 2025). It develops from an imbalance between hepatocellular lipid synthesis/influx and oxidation/export. Our meta-analysis showed that curcumin modulated key lipid-related indicators to correct this imbalance. It significantly reduced serum levels of TC and TG. TC and TG are key drivers of hepatic lipid accumulation (W et al., 2021). Additionally, curcumin lowered LDL cholesterol. LDL is linked to systemic dyslipidemia and MASLD progression (Du et al., 2017). Notably, curcumin uniquely increased HDL cholesterol. HDL plays a critical role in reverse cholesterol transport (Bhale et al., 2024). It removed excess cholesterol from peripheral tissues, including the liver (Diaz and Bielczyk-Maczynska, 2025). This helped reduce atherosclerotic risk for MASLD patients, who often have comorbid metabolic syndrome (Fadaei et al., 2018). These lipid-modulating effects of curcumin were consistent with meta-analyses of curcumin in clinical trials (Mirhafez et al., 2019). Studies have highlighted the crucial role of lipid buildup in the advancement and development of MASLD (Pihl et al., 2005). Hepatic steatosis results from an excess of TG due to the disparity between lipid influx and synthesis on one side, and their β-oxidation and export on the other (Idilman et al., 2016). Furthermore, hepatic steatosis is considered a standalone risk factor for metabolic syndromes like insulin resistance, dyslipidemia, and cardiovascular ailments, showing a stronger correlation with metabolic syndromes than obesity (Idilman et al., 2016). Regulating circulating lipid levels and inhibiting intrahepatic lipid buildup are key in treating hepatic steatosis. Curcumin raised serum HDL levels and reduced serum LDL, TG, and TC levels in high-fat diet conditions (Fabbrini and Magkos, 2015). The accumulation of TG is believed to surpass and impede the oxidative breakdown of FFA (Kim et al., 2014). These conclusions were basically consistent with the outcome for clinical trial meta-analyses. Therefore, the experimental results of our article can withstand scrutiny.

Impaired liver enzyme is a key indicator of hepatocellular damage in MASLD (Grob et al., 2023). Our study showed that curcumin had the effect of reducing liver enzymes. 21 studies on ALT showed that curcumin significantly reduced this marker. This reduction indicates less hepatocyte necrosis and inflammation. The effect size for AST was larger than that for ALT. These results showed that curcumin had the effect of lowering liver enzyme. However, further research is needed to confirm this. Curcumin also reduced body weight and liver index. These changes were likely secondary to reduced hepatic steatosis and improved systemic metabolism. In MASLD models, liver weight is closely tied to intrahepatic fat content. Notably, curcumin had no significant effect on serum insulin. This might be due to the small sample size (only three studies) and high heterogeneity (I^2^ = 96%).

Inflammation and oxidative stress drive MASLD progression from steatosis to MASH and fibrosis (Erceg et al., 2025). Our meta-analysis confirmed that curcumin had anti-inflammatory and antioxidant effects. Curcumin significantly reduced TNF-α and IL-1. These cytokines fuel MASLD: TNF-α promoted liver lipid buildup, while IL-6 accelerated progression to cirrhosis or cancer. Curcumin also reduced the NAS score, proving its biochemical benefits translate to better liver tissue. However, most studies only provided qualitative histology (H&E images), limiting assessment of steatosis/fibrosis regression. Numerous studies have shown that curcumin exhibits anti-inflammatory and antioxidant properties by inhibiting enzymes that produce reactive oxygen species (ROS), such as xanthine dehydrogenase/oxidase, lipoxygenase/cyclooxygenase, and inducible nitric oxide synthase (Li Y. et al., 2023; Lin and Lin-Shiau, 2001). Studies have shown that curcumin reduce liver enzymes and decrease cirrhosis (Afrin R. et al., 2017). Inflammation, oxidative stress, and fat accumulation in the liver are closely associated with the progression of liver and bile duct disorders (Hooijmans et al., 2014). Effective management of inflammation is vital in the treatment of these conditions since hepatitis can advance to cirrhosis or cancer (An et al., 2020; Berasain et al., 2009). This systematic review and meta-analysis aimed to evaluate the antioxidant and anti-inflammatory impacts of curcumin in the management of MASLD. The results revealed that curcumin notably decreased levels of TNF-α and IL-6, which were crucial pro-inflammatory cytokines. The anti-inflammatory impacts of curcumin were revealed through a notable decrease in hepatic and serum levels of TNF-α, which was raised by the MCD diet (Girón-González et al., 2004). Curcumin significantly lowered the hepatic value of IL-6 in C57BL/6 mice given an MCD diet, models illustrating the crucial function of IL-6 in progressing from fatty liver to cirrhosis or cancer (Uchio et al., 2018). Additionally, TNF-α stimulates the buildup of cholesterol in liver cells (Zhang et al., 2023). Elevated TNF-α played a vital part in lipid metabolism and hepatocyte mortality (Ma et al., 2008; Henao-Mejia et al., 2012). TNF-α promoted the accumulation of cholesterol in hepatocytes by increasing absorption through LDL receptors and impeding removal via ABCG1 (Voloshyna et al., 2014). Hepatocytes with excess lipids are susceptible to programmed cell death when TNF-α is present (Pacia et al., 2023). Previous research suggested a significant association between curcumin and reduced TNF-α concentrations (Miura and Ohnishi, 2014). Likewise, our study demonstrated a decline in inflammatory indicators after curcumin treatment. Insulin resistance, a core feature of metabolic syndrome, also served as a crucial pathological basis for diseases such as MASLD (Yang SM. et al., 2023). In rats fed an HFD, significant elevations were observed in liver function indicators such as ALT, AST, blood lipid levels, glucose intolerance, and insulin resistance (Chen et al., 2017). Existing studies have indicated that curcumin exhibits certain potential in improving insulin resistance, and its mechanism of action is closely associated with regulating metabolic pathways, inhibiting inflammatory responses, and alleviating oxidative stress (Li et al., 2024). Moreover, administration of *Lactobacillus* acidophilus probiotics and curcumin to rats with fructose-induced metabolic syndrome improved hormone levels and reduced insulin resistance (Kapar and Ciftci, 2020). Clinical studies have also found that nano-curcumin improves glucose indices, lipid profiles, inflammation in overweight and obese patients with MASLD (Jazayeri-Tehrani et al., 2019).

**FIGURE 22:**
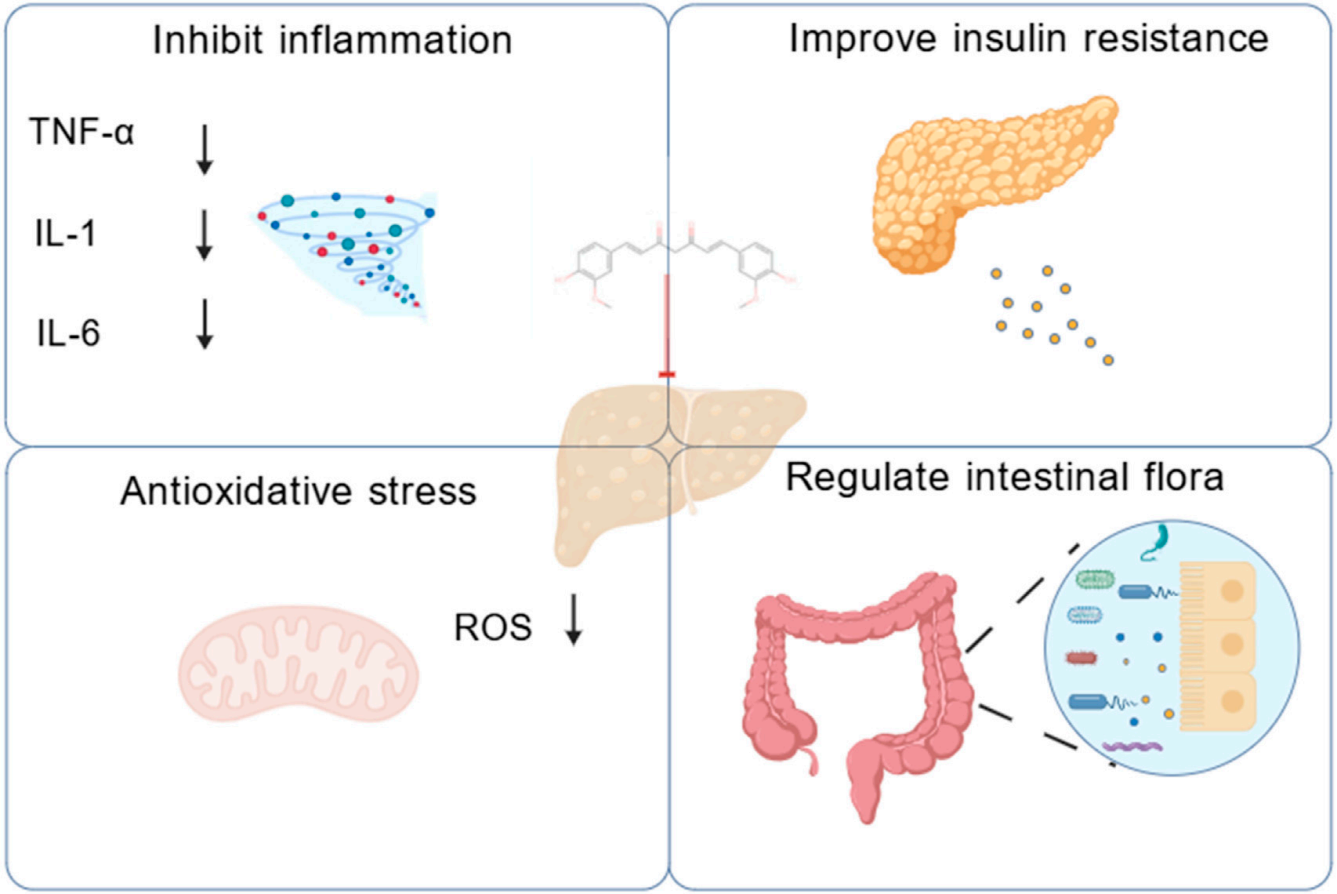
The role and mechanisms of curcumin in the treatment of MASLD.

Intestinal flora imbalance plays a key role in the pathogenesis of MASLD through its metabolites. Restoration of the intestinal microbiota and supplementation of symbiotic bacterial metabolites offer potential therapeutic strategies for this condition (Chen and Vitetta, 2020). Studies have shown that curcumin alleviates bisphenol A (BPA)-induced hepatic steatosis by regulating intestinal flora imbalance and the associated activation of the gut-liver axis (Hong et al., 2022). Curcumin also attenuated ectopic hepatic lipid deposition, improved intestinal barrier integrity, and alleviated metabolic endotoxemia (Feng et al., 2017). Curcumin ameliorated hepatic steatosis and insulin resistance by downregulating the hepatic c-Jun N-terminal kinase 2 (JNK2)/Fork head box protein O1(FOXO1)/B-cell lymphoma 6 (Bcl6) axis and altering the composition and structure of the intestinal flora (Yang et al., 2025). Based on the findings of this review, curcumin showed therapeutic efficacy. It worked in a wide array of MASLD and MASH models. By conducting a comprehensive meta-analysis of the included studies, we developed a mechanistic diagram to illustrate these effects (Figure 22).

High heterogeneity (I^2^ > 50%) was observed across most outcome indicators, a common occurrence in preclinical meta-analyses attributed to variations in experimental designs. To explore the sources of this heterogeneity, we conducted subgroup analyses on key indicators based on animal strain, modeling method, administration route, and curcumin dosage. The results revealed that HDL or LDL heterogeneity was likely associated with curcumin dose. For HDL and LDL, the effect of curcumin at a dose of ≤100 is superior to that at a dose of >100. For ALT, AST, and LDL, duration might serve as a key regulatory factor contributing to their heterogeneity. For AST, ALT, and LDL, the intervention effect with a duration of <8 weeks is significantly better than that with a duration of ≥8 weeks. However, there is high heterogeneity among the studies, so the results need to be interpreted with caution.

Other factors, like animal strain and modeling method, could not explain the heterogeneity. This showed that it was necessary to develop standardized experimental protocols in future preclinical studies. For outcome indicators with an I^2^ > 50%, a random-effects model was used for pooled analysis in this study.

To assess the robustness of the results, we performed sensitivity analyses (removing one study at a time) for all outcome indicators. These findings showed that removing any single study did not significantly alter the overall effect size, confirming that conclusions were stable. Due to significant heterogeneity, a random-effects model was used for the primary meta-analysis to pool the effect sizes. Publication bias was one of the major limitations of meta-analyses. For indicators with ≥10 included studies, we used funnel plots and Egger’s test to detect bias. The results indicated the presence of publication bias in some indicators. The subsequent trim-and-fill analysis demonstrated that after imputing the missing “negative” studies, the direction and significance of the pooled results remained unchanged.

However, this study has several unavoidable limitations. First, some of the included studies failed to provide detailed baseline characteristics. Meanwhile, given the insufficient methodological quality of some studies, the results of this study should be interpreted with caution, and more high-quality studies are needed in the future. The asymmetry of the funnel plot indicated that some small-scale studies with negative results might not have been included in the literature. This suggested that the actual effect of curcumin might be smaller than what was shown in the meta-analysis. Third, leave-one-out sensitivity analysis and trim-and-fill analysis can only reflect the numerical stability of pooled effects, and cannot eliminate the risk of bias. Fourth, the overall methodological quality of the included studies is suboptimal. Furthermore, the study quality assessment results show that 17 studies did not implement blinding in outcome assessment, which may lead to researchers’ subjective judgments interfering with outcome measurement results. Furthermore, the curcumin doses used in the included animal experiments are usually relatively high, which may far exceed the feasible dose for human application. Fifth, there is high heterogeneity among the included studies, mainly resulting from differences in study designs, model construction methods and evaluation criteria for outcome indicators. In addition, animal models differ from human in MASLD, and the known limitations of curcumin (such as low bioavailability in humans) may restrict the direct translational application of the dosing regimen used in this study. Finally, current evidence from *in vitro and in vivo* experiments regarding the efficacy of curcumin in the treatment of MASLD is relatively limited. Therefore, further verification through high-quality clinical studies is required. In the future, priority should be given to advancing large-sample, multi-center, randomized double-blind controlled clinical trials.

## Conclusion

This systematic preclinical evaluation showed that curcumin reduced body weight, liver weight, and other pathological manifestations associated with MASLD. In terms of mechanism of action, curcumin exerted its effects primarily through the following pathways: it significantly reduced hepatic lipid accumulation, specifically reflected in the regulation of lipid indicators such as TC, TG, LDL, and HDL. It reduced insulin resistance. In addition, it inhibited inflammatory responses and oxidative stress. These findings suggested that curcumin might hold potential as a candidate for relevant applications, though this conclusion should be interpreted with caution given the study limitations.
