# Supplementary material for: The therapeutic effect of curcumin in metabolic dysfunction-associated steatotic liver disease: a systematic review and meta-analysis of animal studies
Source: Front Pharmacol. 2025 Nov 24;16:1714245. doi: 10.3389/fphar.2025.1714245 (PMC12682766; doi:10.3389/fphar.2025.1714245)
Supplement: Supplementary file 1 [file Table1.docx]

**Supplementary Table 1 The subgroup analyses of ALT, AST, TC, TG, HDL, LDL and NAS**

| Parameter | Subgroup |  | SMD [95% CI] | I^2^ (%) | *P* for heterogeneity |
| --- | --- | --- | --- | --- | --- |
| ALT | Strain | rat | -3.00 [-3.82, -2.17] | 83 | <0.00001 |
|  |  | mice | -2.90 [-4.51, -1.29] | 85 | <0.00001 |
|  | Administration method | Gavage | -3.09 [-3.81, -2.38] | 81 | <0.00001 |
|  |  | Intraperitoneal injection | -0.27 [-1.34, -0.79] | 83 | <0.00001 |
|  | Treatment dose (mg/kg) | ≤100 | -3.12 [-4.47, -1.78] | 87 | <0.00001 |
|  |  | >100 | -2.89 [-3.72, -2.06] | 79 | <0.00001 |
|  | Duration (week) | <8 | -4.01 [-5.29, -2.72] | 85 | <0.00001 |
|  |  | ≥8 | -2.08 [-2.75, -1.41] | 69 | <0.00001 |
| AST | Strain | rat | -3.50 [-4.56, -2.45] | 87 | <0.00001 |
|  |  | mice | -3.36 [-5.06, -1.65] | 79 | 0.0001 |
|  | Treatment dose (mg/kg) | ≤100 | -4.14 [-5.97, -2.32] | 88 | <0.00001 |
|  |  | >100 | -3.14 [-4.16, -2.12] | 84 | <0.00001 |
|  | Duration (week) | <8 | -4.98 [-6.90, -3.06] | 90 | <0.00001 |
|  |  | ≥8 | -2.38 [-3.02, -1.74] | 59 | <0.00001 |
| TC | Strain | rat | -2.88 [-4.05, -1.71] | 88 | <0.00001 |
|  |  | mice | -3.14 [-5.02, -1.26] | 65 | 0.001 |
|  | Treatment dose (mg/kg) | ≤100 | -2.93 [-3.57, -2.28] | 0 | <0.00001 |
|  |  | >100 | -2.81 [-4.35, -1.27] | 91 | 0.0004 |
|  | Duration (week) | <8 | -3.41 [-5.10, -1.72] | 90 | <0.00001 |
|  |  | ≥8 | -2.32 [-3.16, -1.47] | 56 | <0.00001 |
| TG | Strain | rat | -2.03 [ -2.90, -1.16] | 85 | <0.00001 |
|  |  | mice | -2.97 [-4.76, -1.17] | 85 | 0.001 |
|  | Modeling methods | HFD | -2.35 [-3.18, -1.52] | 85 | <0.00001 |
|  |  | HSFD | -1.37 [-2.29, -0.46] | 84 | 0.003 |
|  |  |  |  |  |  |
|  | Treatment dose (mg/kg) | ≤100 | -3.14 [-5.24, -1.05] | 93 | 0.003 |
|  |  | >100 | -1.93 [-2.44, -1.42] | 51 | <0.00001 |
|  | Duration (week) | <8 | -2.53 [-3.45, -1.61] | 80 | <0.00001 |
|  |  | ≥8 | -1.86 [-3.18, -0.53] | 88 | <0.00001 |
| HDL | Treatment dose (mg/kg) | ≤100 | 2.41 [1.35, 3.47] | 13 | <0.00001 |
|  |  | >100 | 1.06 [0.29, 1.83] | 62 | 0.007 |
|  | Duration (week) | <8 | 1.73 [1.07, 2.38] | 0 | <0.00001 |
|  |  | ≥8 | 1.29 [0.07, 2.51] | 77 | 0.04 |
| LDL | Treatment dose (mg/kg) | ≤100 | -3.06 [-4.42, -1.71] | 35 | <0.00001 |
|  |  | >100 | -1.35 [-2.16, -0.53] | 34 | 0.001 |
|  | Duration (week) | <8 | -3.27 [-4.38, -2.15] | 0 | <0.00001 |
|  |  | ≥8 | -1.26 [-1.92, -0.59] | 9 | 0.0002 |
| NAS | Duration (week) | <8 | -1.11 [-1.64, -0.59] | 0 | <0.00001 |
|  |  | ≥8 | -3.53 [-7.71, 0.65] | 84 | 0.1 |
