# Supplementary material for: The therapeutic effect of curcumin in metabolic dysfunction-associated steatotic liver disease: a systematic review and meta-analysis of animal studies
Source: Front Pharmacol. 2025 Nov 24;16:1714245. doi: 10.3389/fphar.2025.1714245 (PMC12682766; doi:10.3389/fphar.2025.1714245)

Supplementary Figure 1 Forest plot of ALT comparison for subgroup of different dose

Study or Subgroup	Experimental Mean	SD	Total	Control Mean	SD	Total	Weight	Std. Mean Difference IV, Random, 95% CI
**1.4.1 ≤100**								
Abd et al.(2021)	31.5	6.6	10	41.4	0.8	10	5.3%	-2.02 [-3.14, -0.90]
Chang et al.(2021)	60.4	1.8	10	78.8	4.4	10	4.1%	-5.24 [-7.26, -3.23]
Li et al.(2013)	43.67	3.22	12	83	4.36	12	2.8%	-9.91 [-13.08, -6.74]
Lin et al.(2024)	49	5.06	11	75.1	6.33	11	4.6%	-4.38 [-6.04, -2.73]
Mai et al.(2011)	58.85	12.8	10	70.34	11.25	10	5.5%	-0.91 [-1.84, 0.02]
Tong et al.(2021)	1,350	90	8	1,610	160	8	5.1%	-1.89 [-3.13, -0.66]
Vizzutti et al.(2010)	46.3	20.6	6	52	19	8	5.4%	-0.27 [-1.34, 0.79]
Wu et al.(2023)	17.4	4	6	33.4	3.5	6	3.8%	-3.93 [-6.16, -1.70]
Yan et al. (2018)	21.5	4.6	6	67.2	21.1	6	4.5%	-2.76 [-4.52, -1.00]
**Subtotal (95% CI)**			**79**			**81**	**41.1%**	**-3.12 [-4.47, -1.78]**
Heterogeneity: Tau² = 3.46; Chi² = 60.77, df = 8 (P < 0.00001); I² = 87%								
Test for overall effect: Z = 4.56 (P < 0.00001)								
**1.4.2 >100**								
Chen et al.(2015)	44.63	3.74	16	69.33	4.37	40	5.1%	-5.79 [-7.05, -4.54]
Gao et al.(2016)	53.8	6.5	8	100	11	8	3.9%	-4.83 [-7.00, -2.67]
Gu et al.(2024)	71.6	17.2	8	115	25	8	5.1%	-1.91 [-3.15, -0.67]
Hou et al.(2017a)	127.63	21.22	10	203.23	26.25	10	5.0%	-3.03 [-4.40, -1.67]
Hou et al.(2017b)	83.75	16.71	10	131.98	22.7	10	5.2%	-2.32 [-3.51, -1.13]
Shu et al.(2016)	102.2	17.5	9	131.4	19.2	9	5.3%	-1.51 [-2.59, -0.43]
Sun et al.(2021)	48.38	13.32	8	70.25	13.04	8	5.2%	-1.57 [-2.73, -0.41]
Tan et al.(2007)	48	24	10	106	41	14	5.5%	-1.60 [-2.55, -0.65]
Wu et al.(2020)	14.12	4.43	10	24.76	3.46	10	5.1%	-2.56 [-3.81, -1.32]
Xie et al.(2013)	73.5	12.46	6	108	12.94	6	4.6%	-2.51 [-4.17, -0.84]
Xue et al.(2007)	48.36	7.14	10	73.54	11.46	10	5.1%	-2.53 [-3.76, -1.29]
Zhang et al.(2022)	93.4	11.6	10	202	19	10	3.6%	-6.61 [-9.06, -4.16]
**Subtotal (95% CI)**			**115**			**143**	**58.9%**	**-2.89 [-3.72, -2.06]**
Heterogeneity: Tau² = 1.64; Chi² = 52.25, df = 11 (P < 0.00001); I² = 79%								
Test for overall effect: Z = 6.82 (P < 0.00001)								
**Total (95% CI)**			**194**			**224**	**100.0%**	**-2.96 [-3.68, -2.24]**
Heterogeneity: Tau² = 2.22; Chi² = 116.04, df = 20 (P < 0.00001); I² = 83%								
Test for overall effect: Z = 8.05 (P < 0.00001)								
Test for subgroup differences: Chi² = 0.08, df = 1 (P = 0.77), I² = 0%								

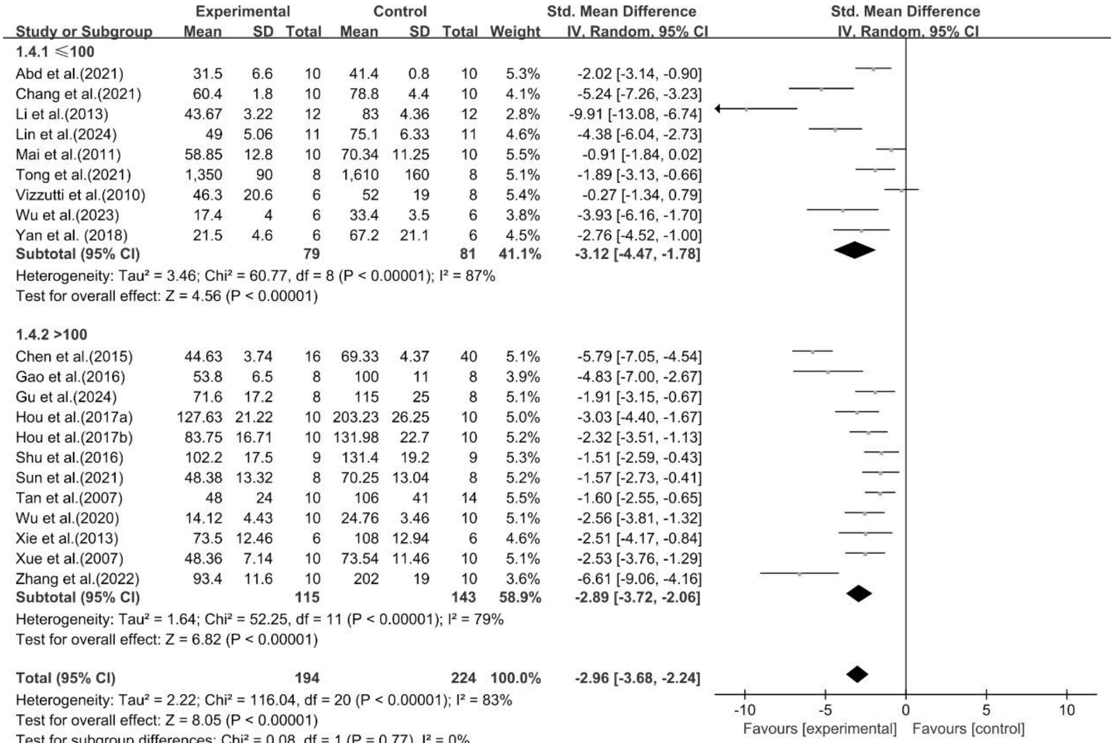

Supplementary Figure 2 Forest plot of AST comparison for subgroup of different dose

Study or Subgroup	Experimental Mean	SD	Total	Control Mean	SD	Total	Weight	Std. Mean Difference IV, Random, 95% CI
**1.7.1 ≤100**								
Abd et al.(2021)	20.2	3.6	10	50.3	1.9	10	3.2%	-10.02 [-13.59, -6.44]
Chang et al.(2021)	139	8	10	182	9	10	5.2%	-4.84 [-6.73, -2.95]
Li et al.(2013)	82.5	7.42	12	122.33	3.51	12	4.8%	-6.63 [-8.83, -4.42]
Lin et al.(2024)	162.7	8.88	11	200.5	8.8	11	5.6%	-4.11 [-5.69, -2.53]
Mai et al.(2011)	66.86	8.77	10	78.68	15.06	10	6.4%	-0.92 [-1.85, 0.01]
Tong et al.(2021)	976	64	8	1,170	120	8	6.0%	-1.91 [-3.15, -0.67]
Wu et al.(2023)	22.7	2.1	6	29.6	2.1	6	5.2%	-3.03 [-4.90, -1.17]
**Subtotal (95% CI)**			**67**			**67**	**36.5%**	**-4.14 [-5.97, -2.32]**
Heterogeneity: Tau² = 5.08; Chi² = 52.02, df = 6 (P < 0.00001); I² = 88%								
Test for overall effect: Z = 4.45 (P < 0.00001)								
**1.7.2 >100**								
Chen et al.(2015)	161.72	8.44	16	259.98	13.77	40	5.6%	-7.74 [-9.33, -6.14]
Gao et al.(2016)	144	9	8	227	25	8	5.1%	-4.18 [-6.11, -2.24]
Gu et al.(2024)	103	34	8	182	43	8	6.0%	-1.93 [-3.17, -0.68]
Hou et al.(2017a)	127.63	21.22	10	203.23	26.25	10	5.9%	-3.03 [-4.40, -1.67]
Hou et al.(2017b)	83.75	16.71	10	131.98	22.7	10	6.1%	-2.32 [-3.51, -1.13]
Shu et al.(2016)	61.2	15.7	9	81.7	11.2	9	6.2%	-1.43 [-2.50, -0.37]
Sun et al.(2021)	140.38	16.24	8	189.63	20.5	8	5.8%	-2.52 [-3.92, -1.11]
Tan et al.(2007)	146	27	10	254	81	14	6.3%	-1.61 [-2.57, -0.66]
Wu et al.(2020)	36.66	3.66	10	51.46	4.47	10	5.7%	-3.47 [-4.96, -1.98]
Xie et al.(2013)	193.83	34.77	6	305.83	64.27	6	5.7%	-2.00 [-3.50, -0.51]
Zhang et al.(2022)	107	25	10	235	20	10	5.0%	-5.42 [-7.48, -3.35]
**Subtotal (95% CI)**			**105**			**133**	**63.5%**	**-3.14 [-4.16, -2.12]**
Heterogeneity: Tau² = 2.44; Chi² = 61.66, df = 10 (P < 0.00001); I² = 84%								
Test for overall effect: Z = 6.02 (P < 0.00001)								
**Total (95% CI)**			**172**			**200**	**100.0%**	**-3.46 [-4.34, -2.58]**
Heterogeneity: Tau² = 2.94; Chi² = 113.69, df = 17 (P < 0.00001); I² = 85%								
Test for overall effect: Z = 7.70 (P < 0.00001)								
Test for subgroup differences: Chi² = 0.89, df = 1 (P = 0.35), I² = 0%								

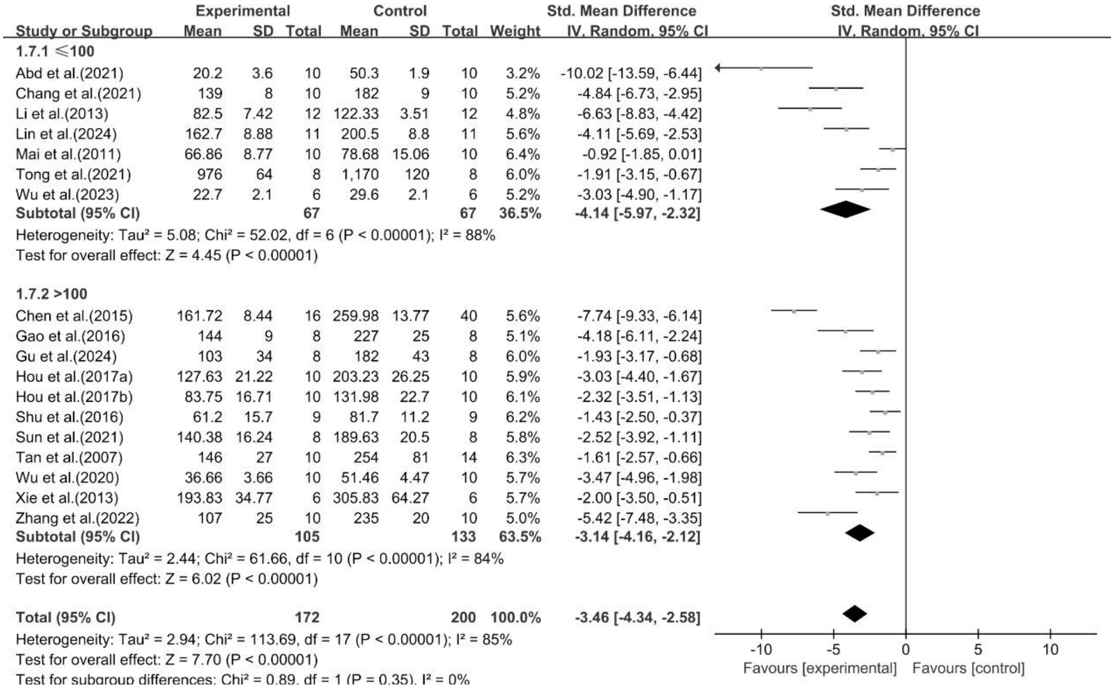

Supplementary Figure 3 Forest plot of TC comparison for subgroup of different dose

Study or Subgroup	Experimental Mean	Experimental SD	Experimental Total	Control Mean	Control SD	Control Total	Weight	Std. Mean Difference IV, Random, 95% CI
**1.10.1 ≤100**								
Afrina et al.(2017)	193.3	8.96	6	258.33	20.31	6	6.8%	-3.82 [-6.01, -1.64]
Li et al.(2013)	1.01	0.06	12	1.4	0.2	12	9.0%	-2.55 [-3.67, -1.43]
Lin et al.(2024)	4.81	0.33	11	7.39	1.22	11	8.8%	-2.78 [-4.01, -1.54]
Mai et al.(2011)	3.78	0.19	10	4.25	0.12	10	8.7%	-2.83 [-4.15, -1.52]
Yan et al. (2018)	2.66	0.26	6	4.23	0.36	6	6.1%	-4.62 [-7.14, -2.09]
**Subtotal (95% CI)**			**45**			**45**	**39.4%**	**-2.93 [-3.57, -2.28]**
Heterogeneity: Tau² = 0.00; Chi² = 2.88, df = 4 (P = 0.58); I² = 0%								
Test for overall effect: Z = 8.88 (P < 0.00001)								
**1.10.2 >100**								
Chen et al.(2015)	1.5	0.06	16	3.02	0.21	40	7.9%	-8.27 [-9.96, -6.58]
Gu et al.(2024)	4.17	0.64	8	6.14	1.39	8	8.9%	-1.72 [-2.92, -0.52]
Shu et al.(2016)	2.3	0.5	9	2.8	0.4	9	9.3%	-1.05 [-2.05, -0.05]
Sun et al.(2021)	1.66	0.24	8	2.1	0.15	8	8.7%	-2.08 [-3.36, -0.79]
Tan et al.(2007)	2.28	0.46	10	4.34	1.7	14	9.4%	-1.48 [-2.42, -0.55]
Wu et al.(2020)	1.18	0.15	10	2.01	0.22	10	7.8%	-4.22 [-5.93, -2.52]
Xie et al.(2013)	1.92	0.42	6	2.47	0.27	6	8.6%	-1.44 [-2.77, -0.11]
**Subtotal (95% CI)**			**67**			**95**	**60.6%**	**-2.81 [-4.35, -1.27]**
Heterogeneity: Tau² = 3.86; Chi² = 64.12, df = 6 (P < 0.00001); I² = 91%								
Test for overall effect: Z = 3.57 (P = 0.0004)								
**Total (95% CI)**			**112**			**140**	**100.0%**	**-2.94 [-3.91, -1.96]**
Heterogeneity: Tau² = 2.42; Chi² = 70.20, df = 11 (P < 0.00001); I² = 84%								
Test for overall effect: Z = 5.89 (P < 0.00001)								
Test for subgroup differences: Chi² = 0.02, df = 1 (P = 0.89), I² = 0%								

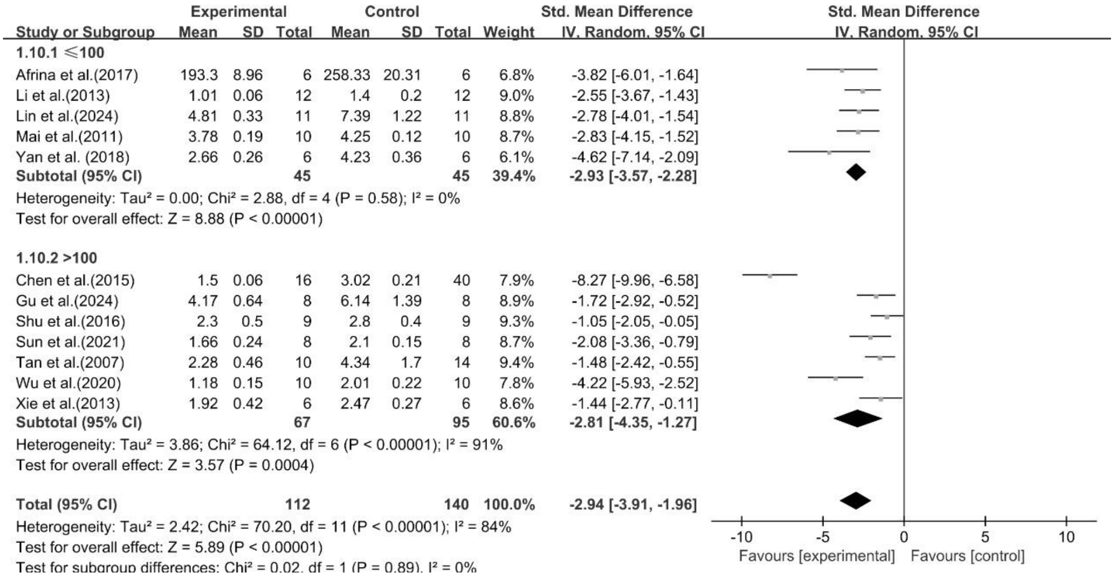

Supplementary Figure 4 Forest plot of TG comparison for subgroup of different dose

Study or Subgroup	Experimental Mean	Experimental SD	Experimental Total	Control Mean	Control SD	Control Total	Weight	Std. Mean Difference IV, Random, 95% CI
**1.13.1 ≤100**								
Afrina et al.(2017)	40.3	11.6	6	43.25	11.6	6	6.4%	-0.23 [-1.37, 0.90]
Chang et al.(2021)	8.15	0.36	10	9.87	0.33	10	5.2%	-4.77 [-6.64, -2.90]
Li et al.(2013)	0.36	0.17	12	0.67	0.14	12	6.7%	-1.92 [-2.92, -0.93]
Lin et al.(2024)	2.81	0.32	11	5.77	0.4	11	3.9%	-7.86 [-10.56, -5.17]
Mai et al.(2011)	2.58	0.27	10	2.27	0.24	10	6.7%	1.16 [0.20, 2.13]
Wu et al.(2023)	0.14	0.04	6	0.28	0.03	6	4.8%	-3.66 [-5.77, -1.54]
Yan et al. (2018)	0.74	0.13	6	2.08	0.22	6	2.9%	-6.85 [-10.37, -3.32]
**Subtotal (95% CI)**			**61**			**61**	**36.5%**	**-3.14 [-5.24, -1.05]**
Heterogeneity: Tau² = 6.98; Chi² = 80.18, df = 6 (P < 0.00001); I² = 93%								
Test for overall effect: Z = 2.94 (P = 0.003)								
**1.13.2 >100**								
Chen et al.(2015)	0.78	0.13	16	1.24	0.23	40	7.0%	-2.19 [-2.91, -1.47]
Gao et al.(2016)	0.18	0.03	8	0.51	0.13	8	5.6%	-3.31 [-4.95, -1.66]
Gu et al.(2024)	1.85	0.2	8	2.4	0.27	8	6.1%	-2.19 [-3.50, -0.88]
Shu et al.(2016)	0.8	0.3	9	1.2	0.4	9	6.6%	-1.08 [-2.08, -0.07]
Sun et al.(2021)	0.63	0.05	8	0.68	0.07	8	6.6%	-0.78 [-1.81, 0.25]
Tan et al.(2007)	0.62	0.18	10	0.92	0.23	14	6.8%	-1.37 [-2.29, -0.46]
Wu et al.(2020)	2.14	0.45	10	3.25	0.44	10	6.3%	-2.39 [-3.59, -1.18]
Xie et al.(2013)	0.82	0.19	6	1.21	0.39	6	6.2%	-1.17 [-2.44, 0.09]
Xue et al.(2007)	2.11	0.32	10	3.33	0.42	10	6.0%	-3.13 [-4.52, -1.74]
Zhang et al.(2022)	67.5	6.8	10	93.2	10.8	10	6.2%	-2.73 [-4.02, -1.44]
**Subtotal (95% CI)**			**95**			**123**	**63.5%**	**-1.93 [-2.44, -1.42]**
Heterogeneity: Tau² = 0.33; Chi² = 18.50, df = 9 (P = 0.03); I² = 51%								
Test for overall effect: Z = 7.40 (P < 0.00001)								
**Total (95% CI)**			**156**			**184**	**100.0%**	**-2.26 [-3.02, -1.49]**
Heterogeneity: Tau² = 2.02; Chi² = 101.77, df = 16 (P < 0.00001); I² = 84%								
Test for overall effect: Z = 5.80 (P < 0.00001)								
Test for subgroup differences: Chi² = 1.22, df = 1 (P = 0.27), I² = 17.7%								

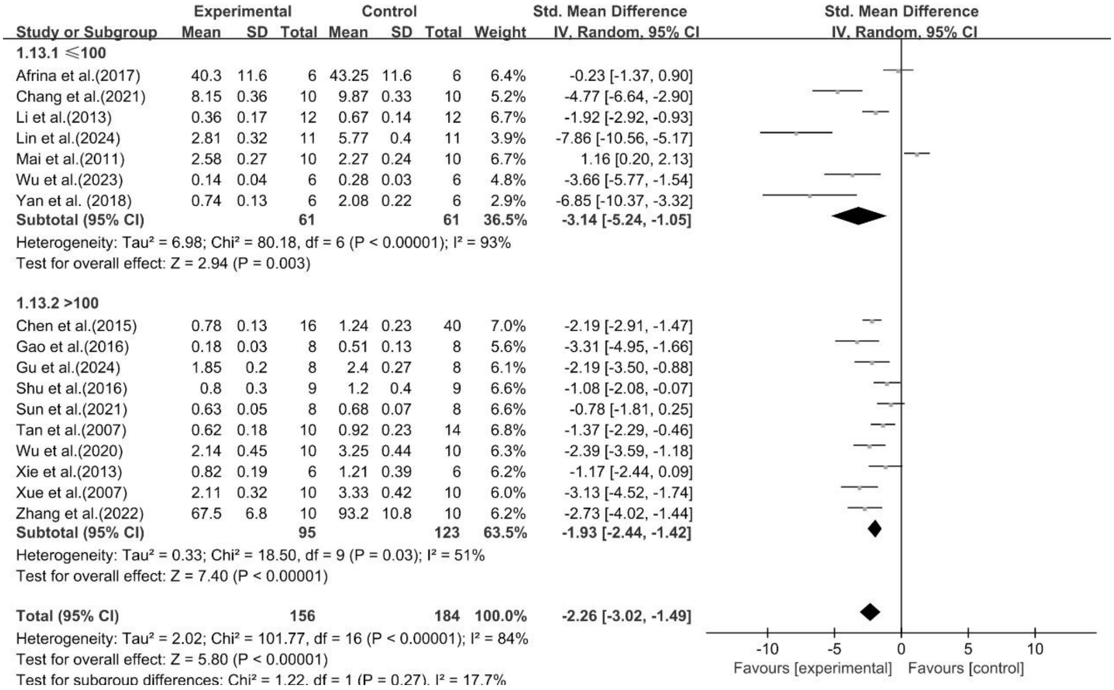

Supplementary Figure 5 Forest plot of HDL comparison for subgroup of different dose

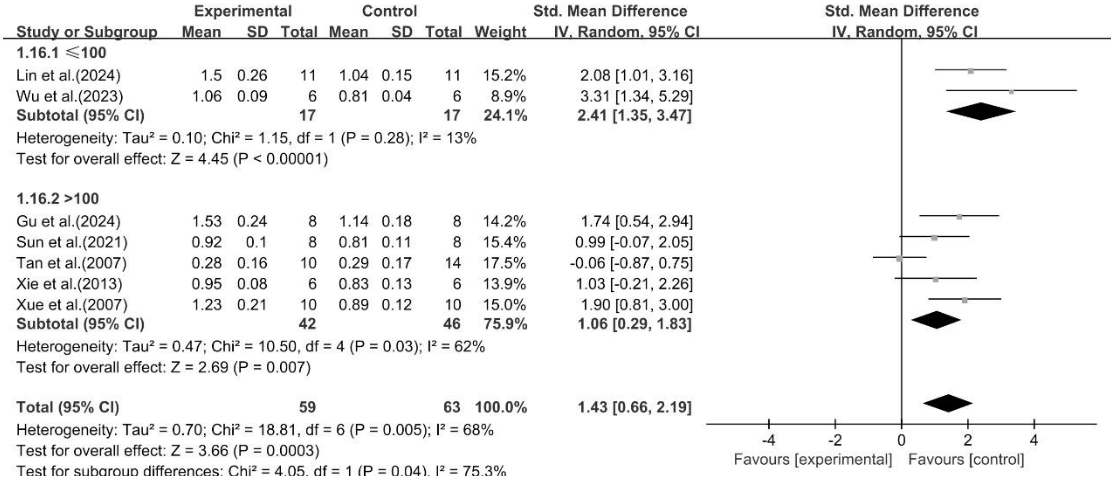

Supplementary Figure 6 Forest plot of LDL comparison for subgroup of different dose

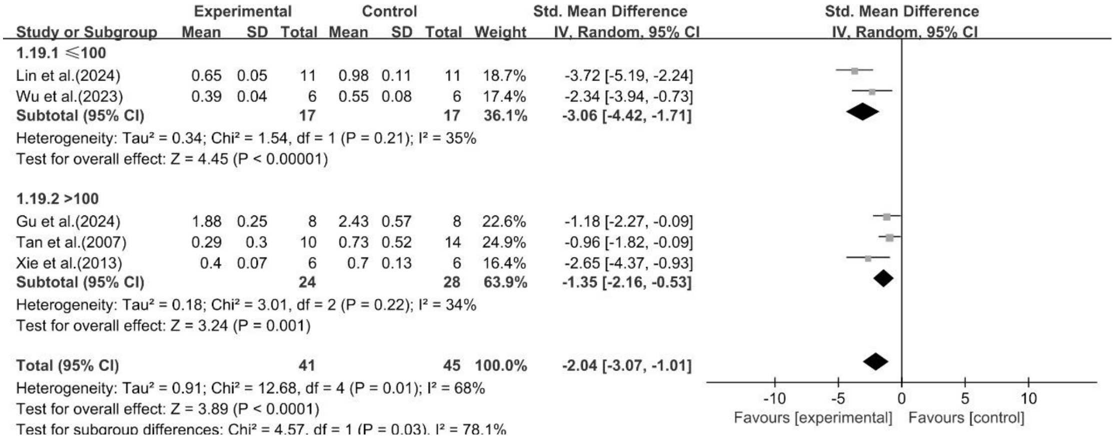

Supplementary Figure 7 Forest plot of NAS comparison for subgroup of different dose

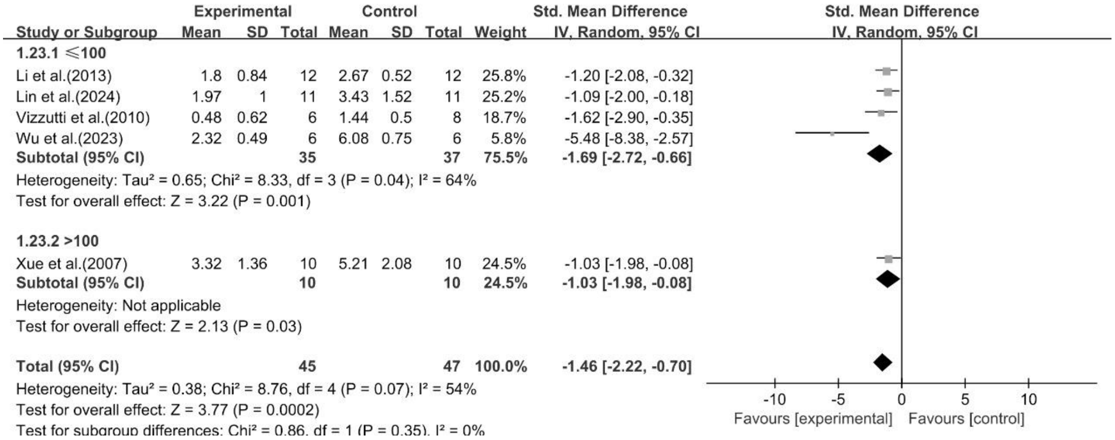

Supplementary Figure 8 Forest plot of ALT comparison for subgroup of different model

Study or Subgroup	Experimental Mean	Experimental SD	Experimental Total	Control Mean	Control SD	Control Total	Weight	Std. Mean Difference IV, Random, 95% CI
**1.2.1 HFD**								
Abd et al.(2021)	31.5	6.6	10	41.4	0.8	10	5.3%	-2.02 [-3.14, -0.90]
Chang et al.(2021)	60.4	1.8	10	78.8	4.4	10	4.1%	-5.24 [-7.26, -3.23]
Chen et al.(2015)	44.63	3.74	16	69.33	4.37	40	5.1%	-5.79 [-7.05, -4.54]
Gao et al.(2016)	53.8	6.5	8	100	11	8	3.9%	-4.83 [-7.00, -2.67]
Gu et al.(2024)	71.6	17.2	8	115	25	8	5.1%	-1.91 [-3.15, -0.67]
Hou et al.(2017a)	127.63	21.22	10	203.23	26.25	10	5.0%	-3.03 [-4.40, -1.67]
Hou et al.(2017b)	83.75	16.71	10	131.98	22.7	10	5.2%	-2.32 [-3.51, -1.13]
Li et al.(2013)	43.67	3.22	12	83	4.36	12	2.8%	-9.91 [-13.08, -6.74]
Lin et al.(2024)	49	5.06	11	75.1	6.33	11	4.6%	-4.38 [-6.04, -2.73]
Mai et al.(2011)	58.85	12.8	10	70.34	11.25	10	5.5%	-0.91 [-1.84, 0.02]
Shu et al.(2016)	102.2	17.5	9	131.4	19.2	9	5.3%	-1.51 [-2.59, -0.43]
Sun et al.(2021)	48.38	13.32	8	70.25	13.04	8	5.2%	-1.57 [-2.73, -0.41]
Wu et al.(2020)	14.12	4.43	10	24.76	3.46	10	5.1%	-2.56 [-3.81, -1.32]
Wu et al.(2023)	17.4	4	6	33.4	3.5	6	3.8%	-3.93 [-6.16, -1.70]
Xie et al.(2013)	73.5	12.46	6	108	12.94	6	4.6%	-2.51 [-4.17, -0.84]
Xue et al.(2007)	48.36	7.14	10	73.54	11.46	10	5.1%	-2.53 [-3.76, -1.29]
Yan et al. (2018)	21.5	4.6	6	67.2	21.1	6	4.5%	-2.76 [-4.52, -1.00]
Zhang et al.(2022)	93.4	11.6	10	202	19	10	3.6%	-6.61 [-9.06, -4.16]
**Subtotal (95% CI)**			**170**			**194**	**84.0%**	**-3.29 [-4.09, -2.49]**
Heterogeneity: Tau² = 2.32; Chi² = 94.18, df = 17 (P < 0.00001); I² = 82%								
Test for overall effect: Z = 8.07 (P < 0.00001)								
**1.2.2 HSFD**								
Tan et al.(2007)	48	24	10	106	41	14	5.5%	-1.60 [-2.55, -0.65]
**Subtotal (95% CI)**			**10**			**14**	**5.5%**	**-1.60 [-2.55, -0.65]**
Heterogeneity: Not applicable								
Test for overall effect: Z = 3.30 (P = 0.0010)								
**1.2.3 MCD**								
Tong et al.(2021)	1,350	90	8	1,610	160	8	5.1%	-1.89 [-3.13, -0.66]
Vizzutti et al.(2010)	46.3	20.6	6	52	19	8	5.4%	-0.27 [-1.34, 0.79]
**Subtotal (95% CI)**			**14**			**16**	**10.5%**	**-1.05 [-2.64, 0.54]**
Heterogeneity: Tau² = 0.97; Chi² = 3.79, df = 1 (P = 0.05); I² = 74%								
Test for overall effect: Z = 1.30 (P = 0.20)								
**Total (95% CI)**			**194**			**224**	**100.0%**	**-2.96 [-3.68, -2.24]**
Heterogeneity: Tau² = 2.22; Chi² = 116.04, df = 20 (P < 0.00001); I² = 83%								
Test for overall effect: Z = 8.05 (P < 0.00001)								
Test for subgroup differences: Chi² = 10.27, df = 2 (P = 0.006), I² = 80.5%								

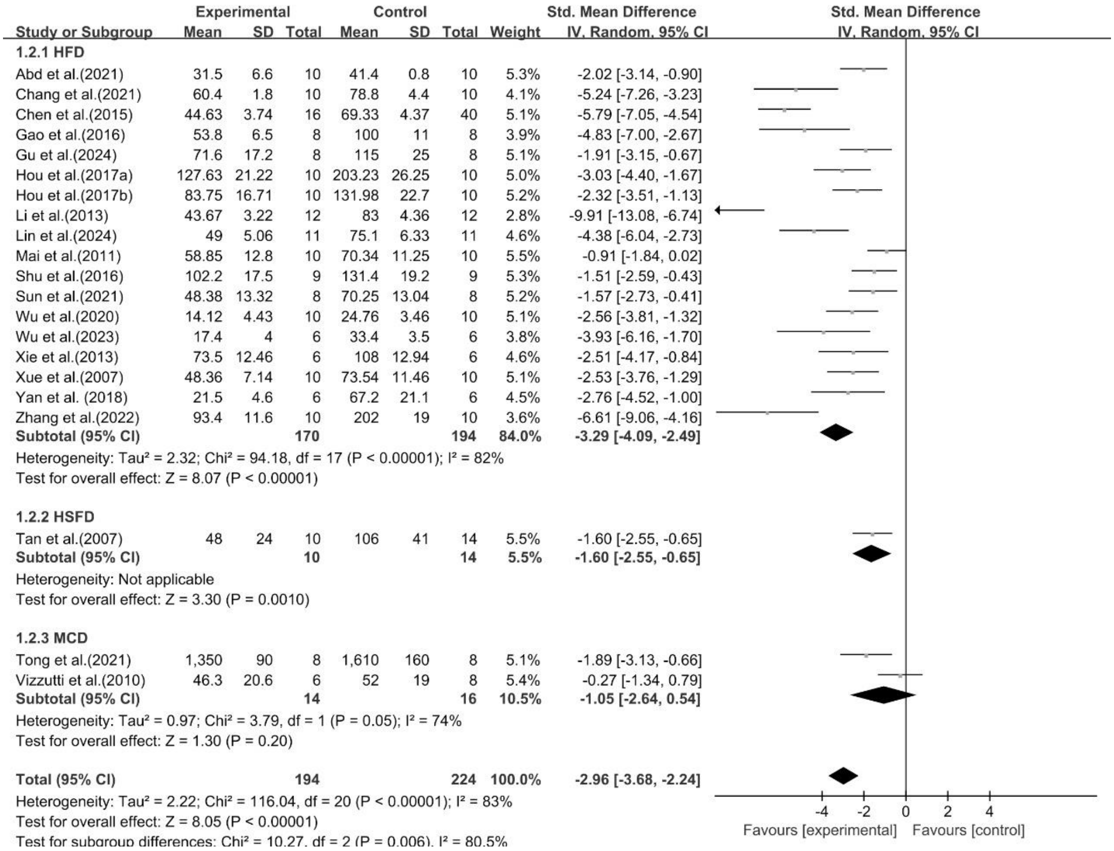

Supplementary Figure 9 Forest plot of AST comparison for subgroup of different model

Study or Subgroup	Experimental Mean	Experimental SD	Experimental Total	Control Mean	Control SD	Control Total	Weight	Std. Mean Difference IV, Random, 95% CI
**1.6.1 HFD**								
Abd et al.(2021)	20.2	3.6	10	50.3	1.9	10	3.2%	-10.02 [-13.59, -6.44]
Chang et al.(2021)	139	8	10	182	9	10	5.2%	-4.84 [-6.73, -2.95]
Chen et al.(2015)	161.72	8.44	16	259.98	13.77	40	5.6%	-7.74 [-9.33, -6.14]
Gao et al.(2016)	144	9	8	227	25	8	5.1%	-4.18 [-6.11, -2.24]
Gu et al.(2024)	103	34	8	182	43	8	6.0%	-1.93 [-3.17, -0.68]
Hou et al.(2017a)	127.63	21.22	10	203.23	26.25	10	5.9%	-3.03 [-4.40, -1.67]
Hou et al.(2017b)	83.75	16.71	10	131.98	22.7	10	6.1%	-2.32 [-3.51, -1.13]
Li et al.(2013)	82.5	7.42	12	122.33	3.51	12	4.8%	-6.63 [-8.83, -4.42]
Lin et al.(2024)	162.7	8.88	11	200.5	8.8	11	5.6%	-4.11 [-5.69, -2.53]
Mai et al.(2011)	66.86	8.77	10	78.68	15.06	10	6.4%	-0.92 [-1.85, 0.01]
Shu et al.(2016)	61.2	15.7	9	81.7	11.2	9	6.2%	-1.43 [-2.50, -0.37]
Sun et al.(2021)	140.38	16.24	8	189.63	20.5	8	5.8%	-2.52 [-3.92, -1.11]
Wu et al.(2020)	36.66	3.66	10	51.46	4.47	10	5.7%	-3.47 [-4.96, -1.98]
Wu et al.(2023)	22.7	2.1	6	29.6	2.1	6	5.2%	-3.03 [-4.90, -1.17]
Xie et al.(2013)	193.83	34.77	6	305.83	64.27	6	5.7%	-2.00 [-3.50, -0.51]
Zhang et al.(2022)	107	25	10	235	20	10	5.0%	-5.42 [-7.48, -3.35]
**Subtotal (95% CI)**			**154**			**178**	**87.6%**	**-3.72 [-4.73, -2.72]**
Heterogeneity: Tau² = 3.43; Chi² = 105.40, df = 15 (P < 0.00001); I² = 86%								
Test for overall effect: Z = 7.29 (P < 0.00001)								
**1.6.2 HSFD**								
Tan et al.(2007)	146	27	10	254	81	14	6.3%	-1.61 [-2.57, -0.66]
**Subtotal (95% CI)**			**10**			**14**	**6.3%**	**-1.61 [-2.57, -0.66]**
Heterogeneity: Not applicable								
Test for overall effect: Z = 3.32 (P = 0.0009)								
**1.6.3 MCD**								
Tong et al.(2021)	976	64	8	1,170	120	8	6.0%	-1.91 [-3.15, -0.67]
**Subtotal (95% CI)**			**8**			**8**	**6.0%**	**-1.91 [-3.15, -0.67]**
Heterogeneity: Not applicable								
Test for overall effect: Z = 3.01 (P = 0.003)								
**Total (95% CI)**			**172**			**200**	**100.0%**	**-3.46 [-4.34, -2.58]**
Heterogeneity: Tau² = 2.94; Chi² = 113.69, df = 17 (P < 0.00001); I² = 85%								
Test for overall effect: Z = 7.70 (P < 0.00001)								
Test for subgroup differences: Chi² = 9.91, df = 2 (P = 0.007), I² = 79.8%								

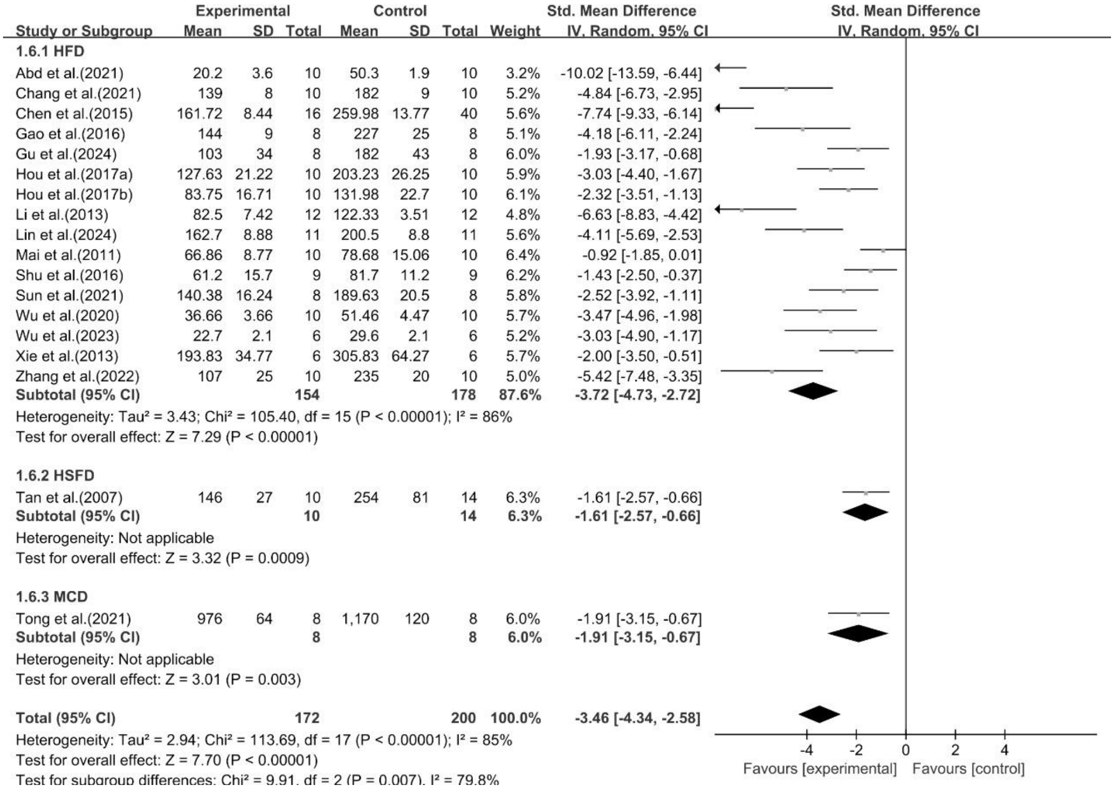

Supplementary Figure 10 Forest plot of TC comparison for subgroup of different model

Study or Subgroup	Experimental Mean	Experimental SD	Experimental Total	Control Mean	Control SD	Control Total	Weight	Std. Mean Difference IV, Random, 95% CI
**1.9.1 HFD**								
Afrina et al.(2017)	193.3	8.96	6	258.33	20.31	6	6.8%	-3.82 [-6.01, -1.64]
Chen et al.(2015)	1.5	0.06	16	3.02	0.21	40	7.9%	-8.27 [-9.96, -6.58]
Gu et al.(2024)	4.17	0.64	8	6.14	1.39	8	8.9%	-1.72 [-2.92, -0.52]
Li et al.(2013)	1.01	0.06	12	1.4	0.2	12	9.0%	-2.55 [-3.67, -1.43]
Lin et al.(2024)	4.81	0.33	11	7.39	1.22	11	8.8%	-2.78 [-4.01, -1.54]
Mai et al.(2011)	3.78	0.19	10	4.25	0.12	10	8.7%	-2.83 [-4.15, -1.52]
Shu et al.(2016)	2.3	0.5	9	2.8	0.4	9	9.3%	-1.05 [-2.05, -0.05]
Sun et al.(2021)	1.66	0.24	8	2.1	0.15	8	8.7%	-2.08 [-3.36, -0.79]
Wu et al.(2020)	1.18	0.15	10	2.01	0.22	10	7.8%	-4.22 [-5.93, -2.52]
Xie et al.(2013)	1.92	0.42	6	2.47	0.27	6	8.6%	-1.44 [-2.77, -0.11]
Yan et al. (2018)	2.66	0.26	6	4.23	0.36	6	6.1%	-4.62 [-7.14, -2.09]
**Subtotal (95% CI)**			**102**			**126**	**90.6%**	**-3.10 [-4.17, -2.02]**
Heterogeneity: Tau² = 2.71; Chi² = 65.31, df = 10 (P < 0.00001); I² = 85%								
Test for overall effect: Z = 5.65 (P < 0.00001)								
**1.9.2 HSFD**								
Tan et al.(2007)	2.28	0.46	10	4.34	1.7	14	9.4%	-1.48 [-2.42, -0.55]
**Subtotal (95% CI)**			**10**			**14**	**9.4%**	**-1.48 [-2.42, -0.55]**
Heterogeneity: Not applicable								
Test for overall effect: Z = 3.12 (P = 0.002)								
**Total (95% CI)**			**112**			**140**	**100.0%**	**-2.94 [-3.91, -1.96]**
Heterogeneity: Tau² = 2.42; Chi² = 70.20, df = 11 (P < 0.00001); I² = 84%								
Test for overall effect: Z = 5.89 (P < 0.00001)								
Test for subgroup differences: Chi² = 4.94, df = 1 (P = 0.03), I² = 79.7%								

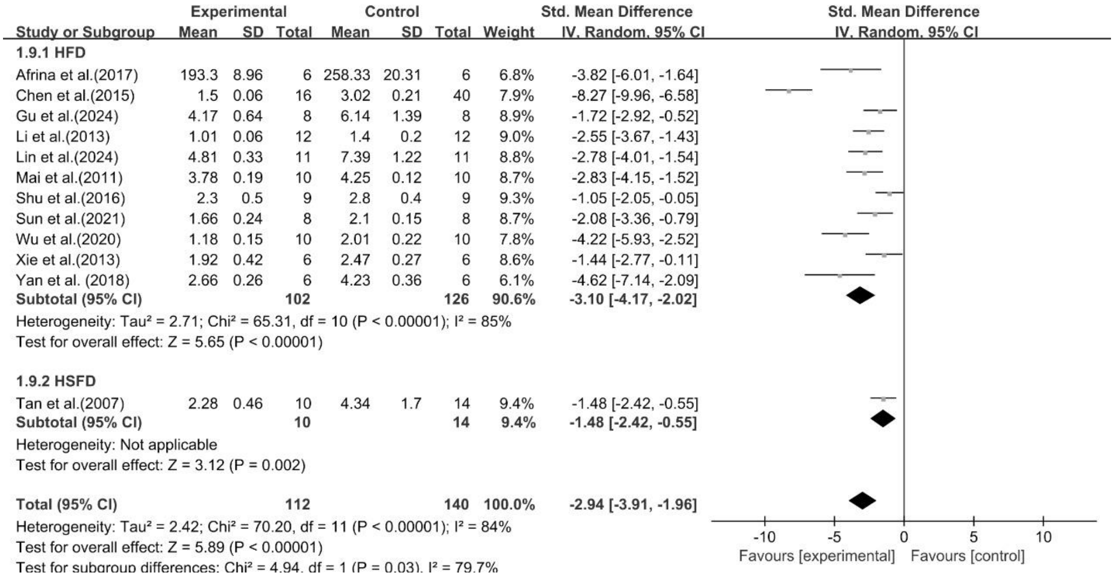

Supplementary Figure 11 Forest plot of TG comparison for subgroup of different model

Study or Subgroup	Experimental Mean	Experimental SD	Experimental Total	Control Mean	Control SD	Control Total	Weight	Std. Mean Difference IV, Random, 95% CI
**1.12.1 HFD**								
Afrina et al.(2017)	40.3	11.6	6	43.25	11.6	6	6.4%	-0.23 [-1.37, 0.90]
Chang et al.(2021)	8.15	0.36	10	9.87	0.33	10	5.2%	-4.77 [-6.64, -2.90]
Chen et al.(2015)	0.78	0.13	16	1.24	0.23	40	7.0%	-2.19 [-2.91, -1.47]
Gao et al.(2016)	0.18	0.03	8	0.51	0.13	8	5.6%	-3.31 [-4.95, -1.66]
Gu et al.(2024)	1.85	0.2	8	2.4	0.27	8	6.1%	-2.19 [-3.50, -0.88]
Li et al.(2013)	0.36	0.17	12	0.67	0.14	12	6.7%	-1.92 [-2.92, -0.93]
Lin et al.(2024)	2.81	0.32	11	5.77	0.4	11	3.9%	-7.86 [-10.56, -5.17]
Mai et al.(2011)	2.58	0.27	10	2.27	0.24	10	6.7%	1.16 [0.20, 2.13]
Shu et al.(2016)	0.8	0.3	9	1.2	0.4	9	6.6%	-1.08 [-2.08, -0.07]
Sun et al.(2021)	0.63	0.05	8	0.68	0.07	8	6.6%	-0.78 [-1.81, 0.25]
Wu et al.(2020)	2.14	0.45	10	3.25	0.44	10	6.3%	-2.39 [-3.59, -1.18]
Wu et al.(2023)	0.14	0.04	6	0.28	0.03	6	4.8%	-3.66 [-5.77, -1.54]
Xie et al.(2013)	0.82	0.19	6	1.21	0.39	6	6.2%	-1.17 [-2.44, 0.09]
Xue et al.(2007)	2.11	0.32	10	3.33	0.42	10	6.0%	-3.13 [-4.52, -1.74]
Yan et al. (2018)	0.74	0.13	6	2.08	0.22	6	2.9%	-6.85 [-10.37, -3.32]
Zhang et al.(2022)	67.5	6.8	10	93.2	10.8	10	6.2%	-2.73 [-4.02, -1.44]
**Subtotal (95% CI)**			**146**			**170**	**93.2%**	**-2.35 [-3.18, -1.52]**
Heterogeneity: Tau² = 2.27; Chi² = 101.22, df = 15 (P < 0.00001); I² = 85%								
Test for overall effect: Z = 5.54 (P < 0.00001)								
**1.12.2 HSFD**								
Tan et al.(2007)	0.62	0.18	10	0.92	0.23	14	6.8%	-1.37 [-2.29, -0.46]
**Subtotal (95% CI)**			**10**			**14**	**6.8%**	**-1.37 [-2.29, -0.46]**
Heterogeneity: Not applicable								
Test for overall effect: Z = 2.94 (P = 0.003)								
**Total (95% CI)**			**156**			**184**	**100.0%**	**-2.26 [-3.02, -1.49]**
Heterogeneity: Tau² = 2.02; Chi² = 101.77, df = 16 (P < 0.00001); I² = 84%								
Test for overall effect: Z = 5.80 (P < 0.00001)								
Test for subgroup differences: Chi² = 2.38, df = 1 (P = 0.12), I² = 58.0%								

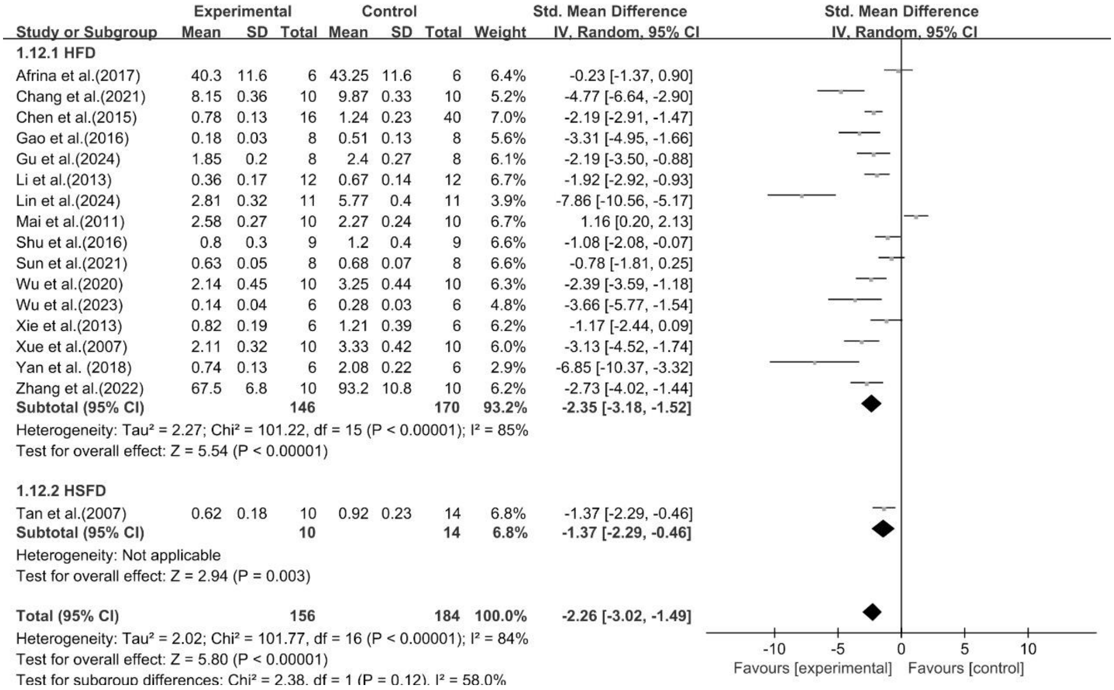

Supplementary Figure 12 Forest plot of HDL comparison for subgroup of different model

Study or Subgroup	Experimental Mean	Experimental SD	Experimental Total	Control Mean	Control SD	Control Total	Weight	Std. Mean Difference IV, Random, 95% CI
**1.15.1 HFD**								
Gu et al.(2024)	1.53	0.24	8	1.14	0.18	8	14.2%	1.74 [0.54, 2.94]
Lin et al.(2024)	1.5	0.26	11	1.04	0.15	11	15.2%	2.08 [1.01, 3.16]
Sun et al.(2021)	0.92	0.1	8	0.81	0.11	8	15.4%	0.99 [-0.07, 2.05]
Wu et al.(2023)	1.06	0.09	6	0.81	0.04	6	8.9%	3.31 [1.34, 5.29]
Xie et al.(2013)	0.95	0.08	6	0.83	0.13	6	13.9%	1.03 [-0.21, 2.26]
Xue et al.(2007)	1.23	0.21	10	0.89	0.12	10	15.0%	1.90 [0.81, 3.00]
**Subtotal (95% CI)**			**49**			**49**	**82.5%**	**1.68 [1.14, 2.22]**
Heterogeneity: Tau² = 0.08; Chi² = 6.05, df = 5 (P = 0.30); I² = 17%								
Test for overall effect: Z = 6.08 (P < 0.00001)								
**1.15.2 HSFD**								
Tan et al.(2007)	0.28	0.16	10	0.29	0.17	14	17.5%	-0.06 [-0.87, 0.75]
**Subtotal (95% CI)**			**10**			**14**	**17.5%**	**-0.06 [-0.87, 0.75]**
Heterogeneity: Not applicable								
Test for overall effect: Z = 0.14 (P = 0.89)								
**Total (95% CI)**			**59**			**63**	**100.0%**	**1.43 [0.66, 2.19]**
Heterogeneity: Tau² = 0.70; Chi² = 18.81, df = 6 (P = 0.005); I² = 68%								
Test for overall effect: Z = 3.66 (P = 0.0003)								
Test for subgroup differences: Chi² = 12.20, df = 1 (P = 0.0005), I² = 91.8%								

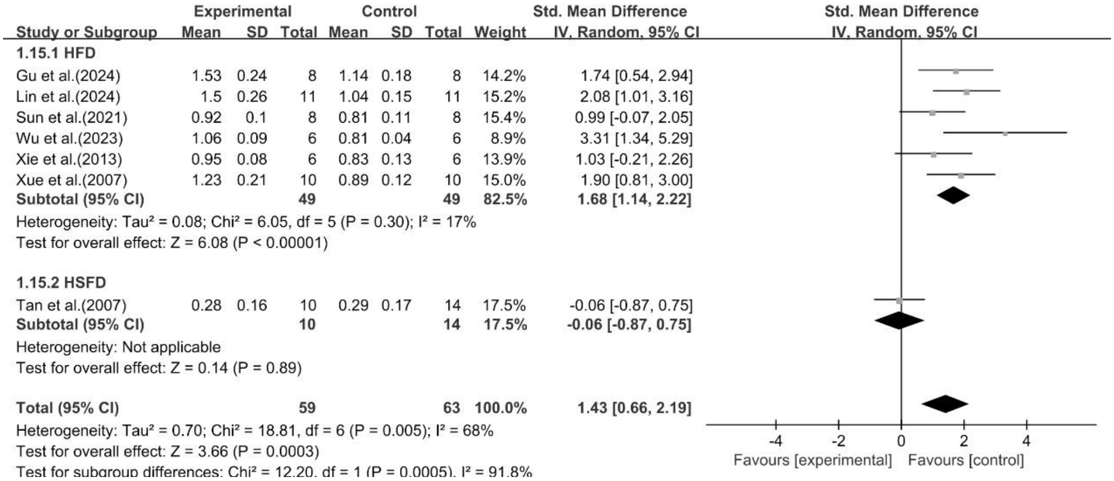

Supplementary Figure 13 Forest plot of LDL comparison for subgroup of different model

Study or Subgroup	Experimental Mean	Experimental SD	Experimental Total	Control Mean	Control SD	Control Total	Weight	Std. Mean Difference IV, Random, 95% CI
**1.18.1 HFD**								
Gu et al.(2024)	1.88	0.25	8	2.43	0.57	8	22.6%	-1.18 [-2.27, -0.09]
Lin et al.(2024)	0.65	0.05	11	0.98	0.11	11	18.7%	-3.72 [-5.19, -2.24]
Wu et al.(2023)	0.39	0.04	6	0.55	0.08	6	17.4%	-2.34 [-3.94, -0.73]
Xie et al.(2013)	0.4	0.07	6	0.7	0.13	6	16.4%	-2.65 [-4.37, -0.93]
**Subtotal (95% CI)**			**31**			**31**	**75.1%**	**-2.40 [-3.56, -1.23]**
Heterogeneity: Tau² = 0.85; Chi² = 7.73, df = 3 (P = 0.05); I² = 61%								
Test for overall effect: Z = 4.04 (P < 0.0001)								
**1.18.2 HSFD**								
Tan et al.(2007)	0.29	0.3	10	0.73	0.52	14	24.9%	-0.96 [-1.82, -0.09]
**Subtotal (95% CI)**			**10**			**14**	**24.9%**	**-0.96 [-1.82, -0.09]**
Heterogeneity: Not applicable								
Test for overall effect: Z = 2.17 (P = 0.03)								
**Total (95% CI)**			**41**			**45**	**100.0%**	**-2.04 [-3.07, -1.01]**
Heterogeneity: Tau² = 0.91; Chi² = 12.68, df = 4 (P = 0.01); I² = 68%								
Test for overall effect: Z = 3.89 (P < 0.0001)								
Test for subgroup differences: Chi² = 3.79, df = 1 (P = 0.05), I² = 73.6%								

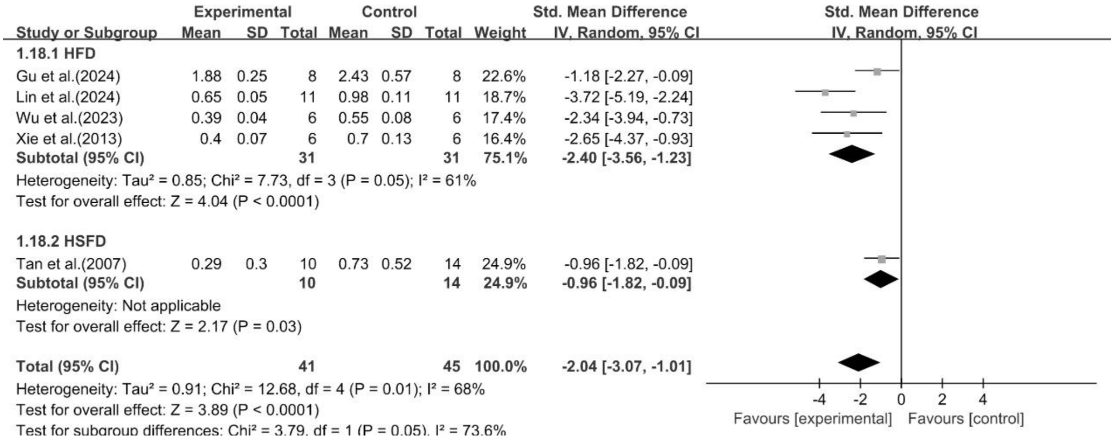

Supplementary Figure 14 Forest plot of NAS comparison for subgroup of different model

Study or Subgroup	Experimental Mean	Experimental SD	Experimental Total	Control Mean	Control SD	Control Total	Weight	Std. Mean Difference IV, Random, 95% CI
**1.21.1 HFD**								
Li et al.(2013)	1.8	0.84	12	2.67	0.52	12	25.8%	-1.20 [-2.08, -0.32]
Lin et al.(2024)	1.97	1	11	3.43	1.52	11	25.2%	-1.09 [-2.00, -0.18]
Wu et al.(2023)	2.32	0.49	6	6.08	0.75	6	5.8%	-5.48 [-8.38, -2.57]
Xue et al.(2007)	3.32	1.36	10	5.21	2.08	10	24.5%	-1.03 [-1.98, -0.08]
**Subtotal (95% CI)**			**39**			**39**	**81.3%**	**-1.48 [-2.44, -0.53]**
Heterogeneity: Tau² = 0.56; Chi² = 8.48, df = 3 (P = 0.04); I² = 65%								
Test for overall effect: Z = 3.05 (P = 0.002)								
**1.21.2 MCD**								
Vizzutti et al.(2010)	0.48	0.62	6	1.44	0.5	8	18.7%	-1.62 [-2.90, -0.35]
**Subtotal (95% CI)**			**6**			**8**	**18.7%**	**-1.62 [-2.90, -0.35]**
Heterogeneity: Not applicable								
Test for overall effect: Z = 2.50 (P = 0.01)								
**Total (95% CI)**			**45**			**47**	**100.0%**	**-1.46 [-2.22, -0.70]**
Heterogeneity: Tau² = 0.38; Chi² = 8.76, df = 4 (P = 0.07); I² = 54%								
Test for overall effect: Z = 3.77 (P = 0.0002)								
Test for subgroup differences: Chi² = 0.03, df = 1 (P = 0.86), I² = 0%								

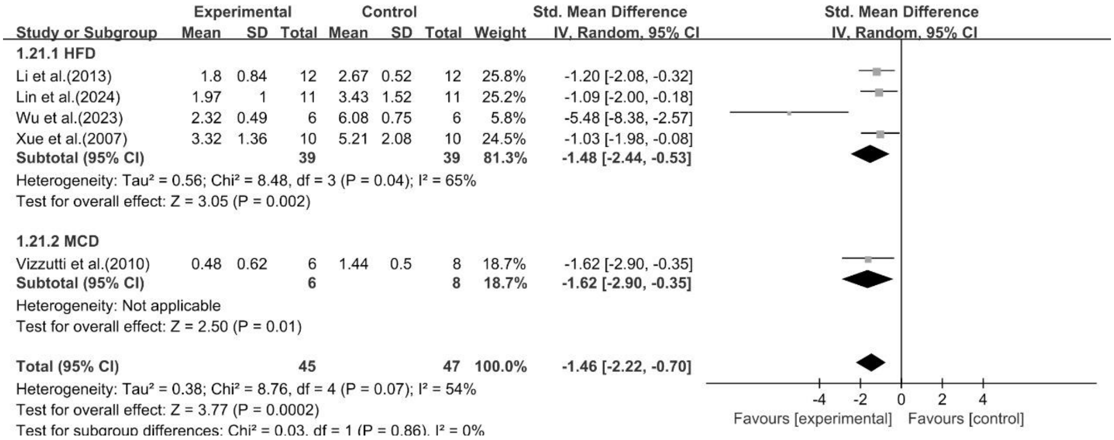

Supplementary Figure 15 Forest plot of ALT comparison for subgroup of different strain

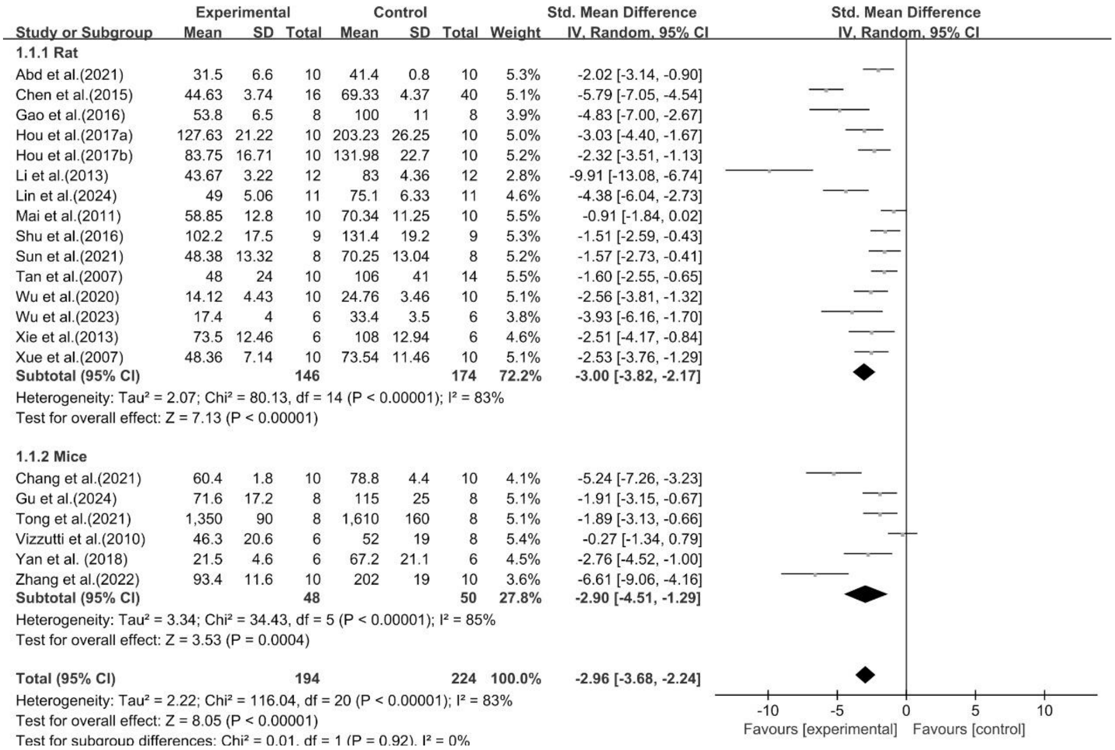

Supplementary Figure 16 Forest plot of AST comparison for subgroup of different strain

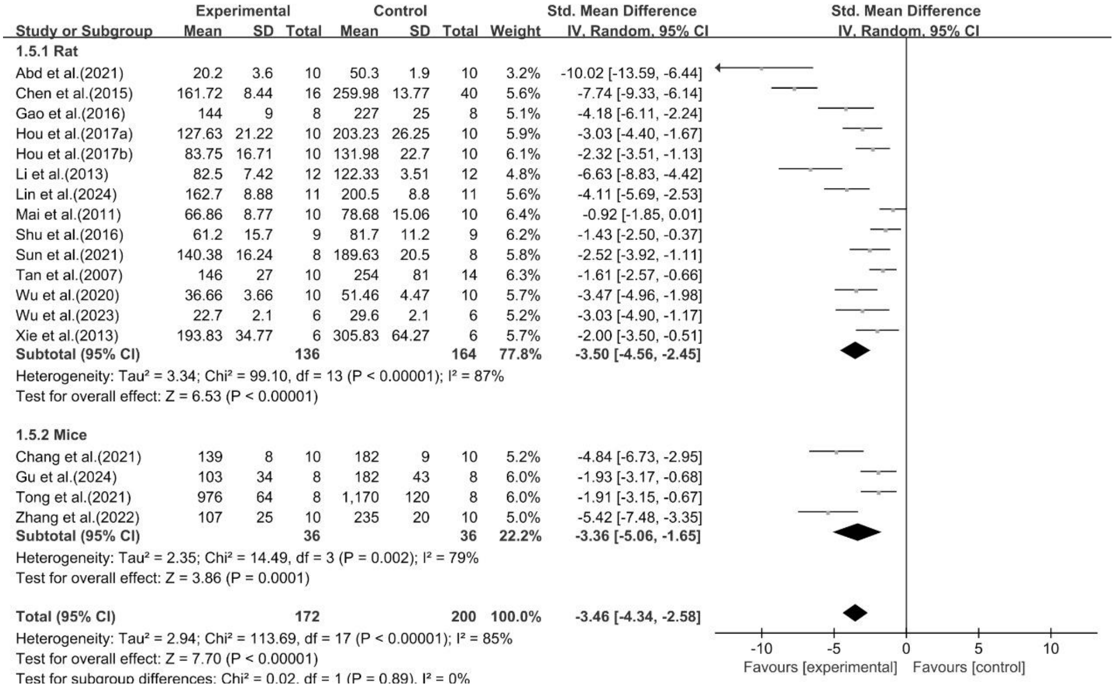

Supplementary Figure 17 Forest plot of TC comparison for subgroup of different strain

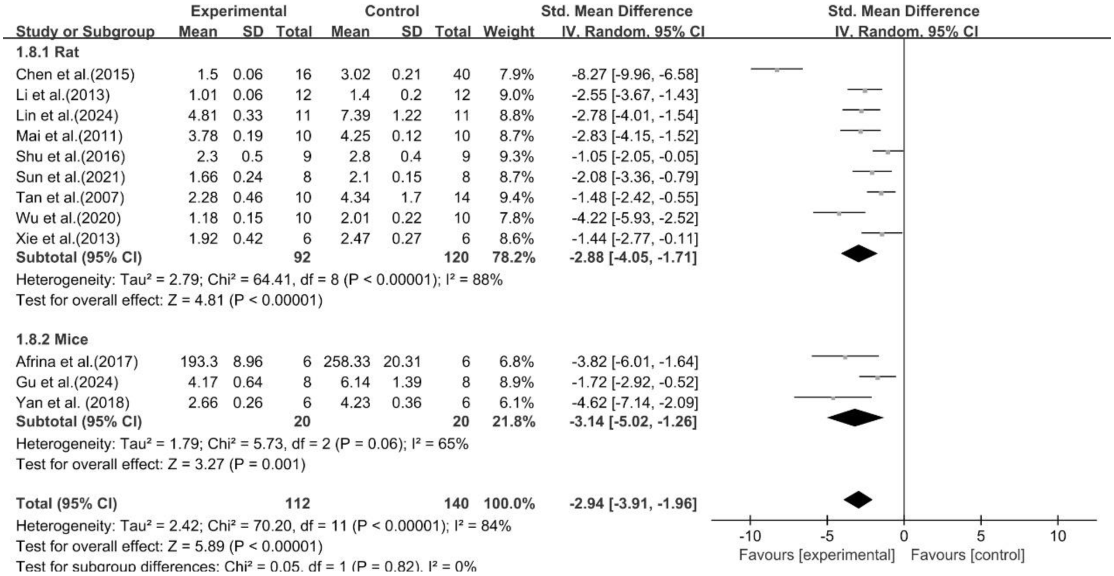

Supplementary Figure 18 Forest plot of TG comparison for subgroup of different strain

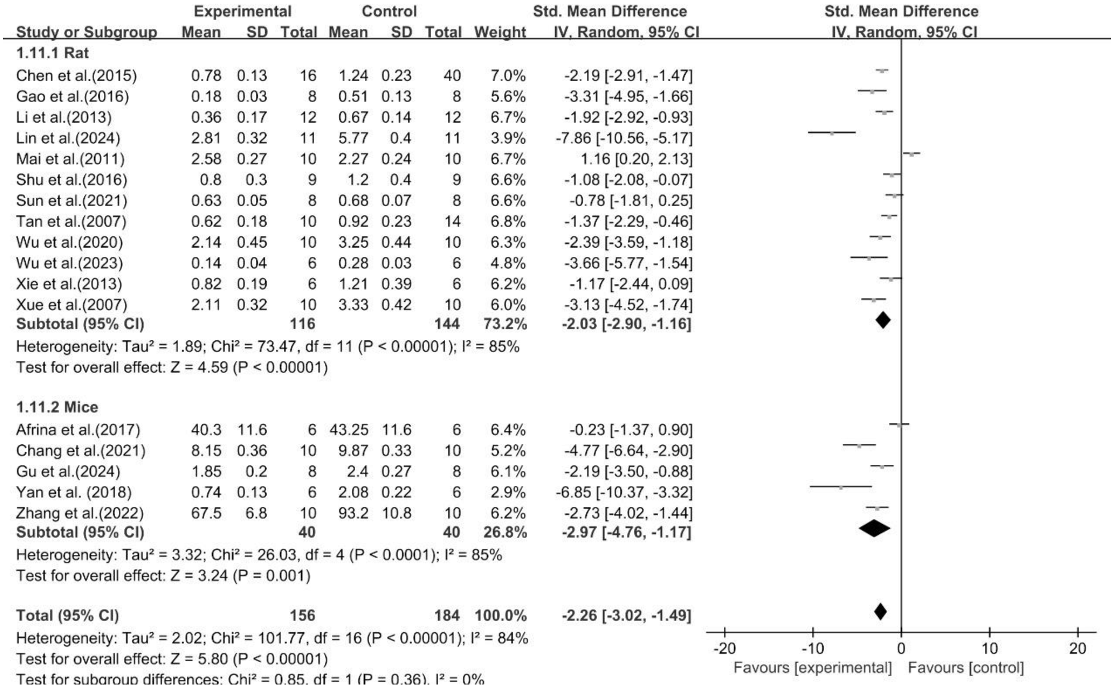

Supplementary Figure 19 Forest plot of HDL comparison for subgroup of different strain

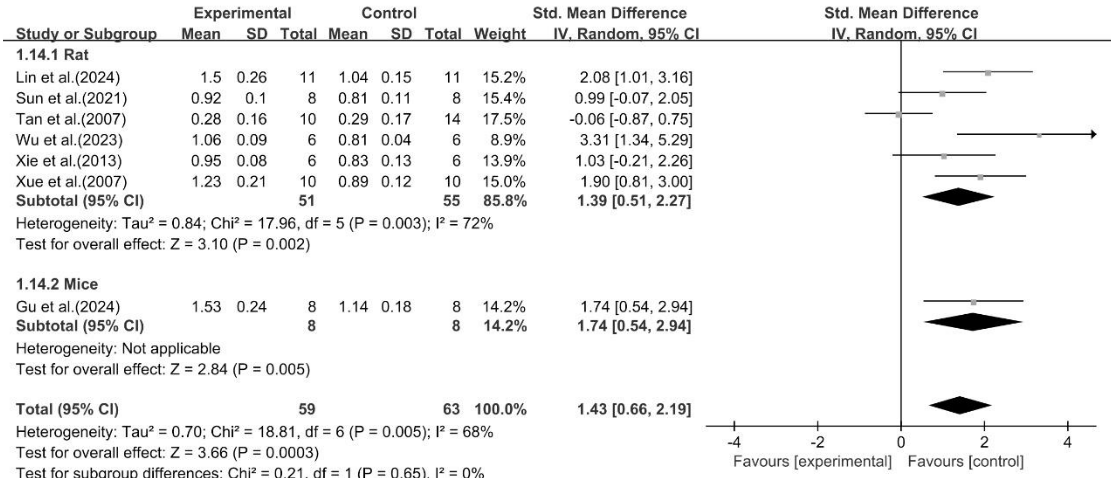

Supplementary Figure 20 Forest plot of LDL comparison for subgroup of different strain

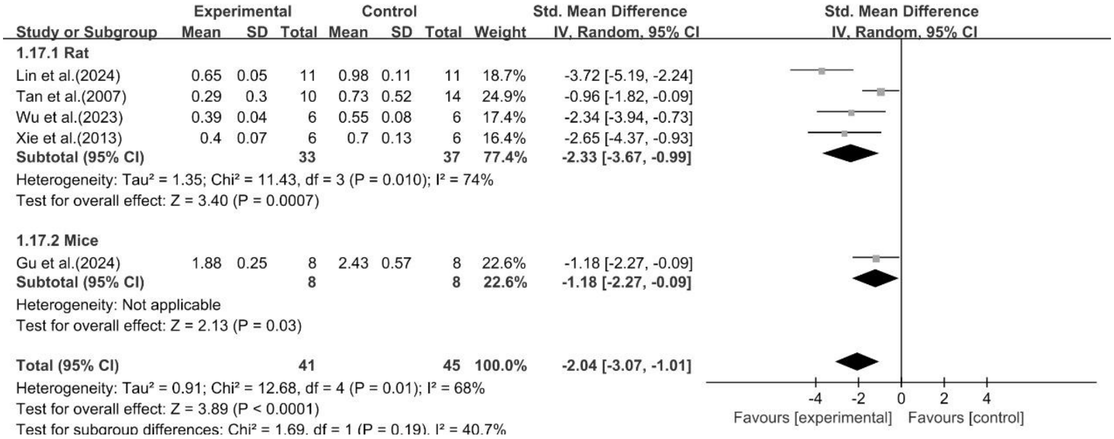

Supplementary Figure 21 Forest plot of NAS comparison for subgroup of different strain

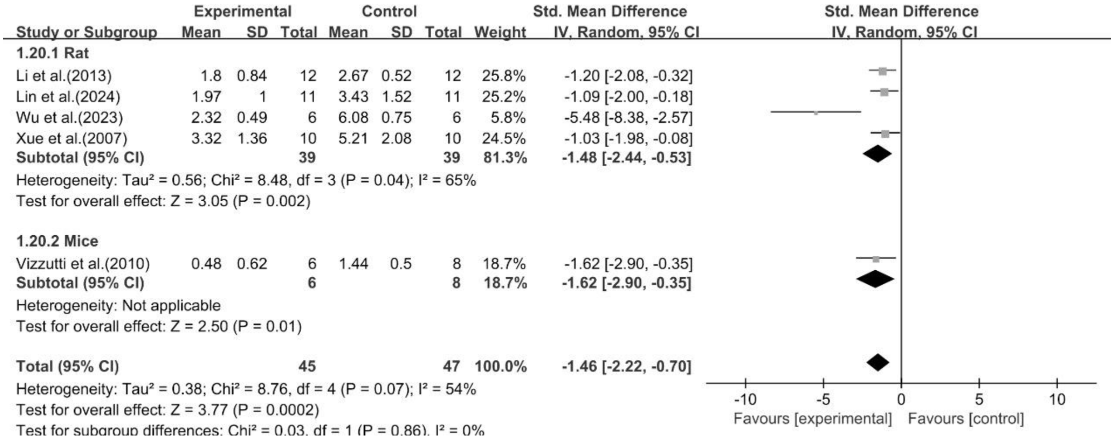

Supplementary Figure 22 Forest plot of ALT comparison for subgroup of different types of administration

Study or Subgroup	Experimental Mean	Experimental SD	Experimental Total	Control Mean	Control SD	Control Total	Weight	Std. Mean Difference IV, Random, 95% CI
**1.3.1 Gavage**								
Abd et al.(2021)	31.5	6.6	10	41.4	0.8	10	5.3%	-2.02 [-3.14, -0.90]
Chang et al.(2021)	60.4	1.8	10	78.8	4.4	10	4.1%	-5.24 [-7.26, -3.23]
Chen et al.(2015)	44.63	3.74	16	69.33	4.37	40	5.1%	-5.79 [-7.05, -4.54]
Gao et al.(2016)	53.8	6.5	8	100	11	8	3.9%	-4.83 [-7.00, -2.67]
Gu et al.(2024)	71.6	17.2	8	115	25	8	5.1%	-1.91 [-3.15, -0.67]
Hou et al.(2017a)	127.63	21.22	10	203.23	26.25	10	5.0%	-3.03 [-4.40, -1.67]
Hou et al.(2017b)	83.75	16.71	10	131.98	22.7	10	5.2%	-2.32 [-3.51, -1.13]
Li et al.(2013)	43.67	3.22	12	83	4.36	12	2.8%	-9.91 [-13.08, -6.74]
Lin et al.(2024)	49	5.06	11	75.1	6.33	11	4.6%	-4.38 [-6.04, -2.73]
Mai et al.(2011)	58.85	12.8	10	70.34	11.25	10	5.5%	-0.91 [-1.84, 0.02]
Shu et al.(2016)	102.2	17.5	9	131.4	19.2	9	5.3%	-1.51 [-2.59, -0.43]
Sun et al.(2021)	48.38	13.32	8	70.25	13.04	8	5.2%	-1.57 [-2.73, -0.41]
Tan et al.(2007)	48	24	10	106	41	14	5.5%	-1.60 [-2.55, -0.65]
Tong et al.(2021)	1,350	90	8	1,610	160	8	5.1%	-1.89 [-3.13, -0.66]
Wu et al.(2020)	14.12	4.43	10	24.76	3.46	10	5.1%	-2.56 [-3.81, -1.32]
Wu et al.(2023)	17.4	4	6	33.4	3.5	6	3.8%	-3.93 [-6.16, -1.70]
Xie et al.(2013)	73.5	12.46	6	108	12.94	6	4.6%	-2.51 [-4.17, -0.84]
Xue et al.(2007)	48.36	7.14	10	73.54	11.46	10	5.1%	-2.53 [-3.76, -1.29]
Yan et al. (2018)	21.5	4.6	6	67.2	21.1	6	4.5%	-2.76 [-4.52, -1.00]
Zhang et al.(2022)	93.4	11.6	10	202	19	10	3.6%	-6.61 [-9.06, -4.16]
**Subtotal (95% CI)**			**188**			**216**	**94.6%**	**-3.09 [-3.81, -2.38]**
Heterogeneity: Tau² = 2.03; Chi² = 99.89, df = 19 (P < 0.00001); I² = 81%								
Test for overall effect: Z = 8.50 (P < 0.00001)								
**1.3.2 Intraperitoneal injection**								
Vizzutti et al.(2010)	46.3	20.6	6	52	19	8	5.4%	-0.27 [-1.34, 0.79]
**Subtotal (95% CI)**			**6**			**8**	**5.4%**	**-0.27 [-1.34, 0.79]**
Heterogeneity: Not applicable								
Test for overall effect: Z = 0.50 (P = 0.62)								
**Total (95% CI)**			**194**			**224**	**100.0%**	**-2.96 [-3.68, -2.24]**
Heterogeneity: Tau² = 2.22; Chi² = 116.04, df = 20 (P < 0.00001); I² = 83%								
Test for overall effect: Z = 8.05 (P < 0.00001)								
Test for subgroup differences: Chi² = 18.61, df = 1 (P < 0.0001), I² = 94.6%								

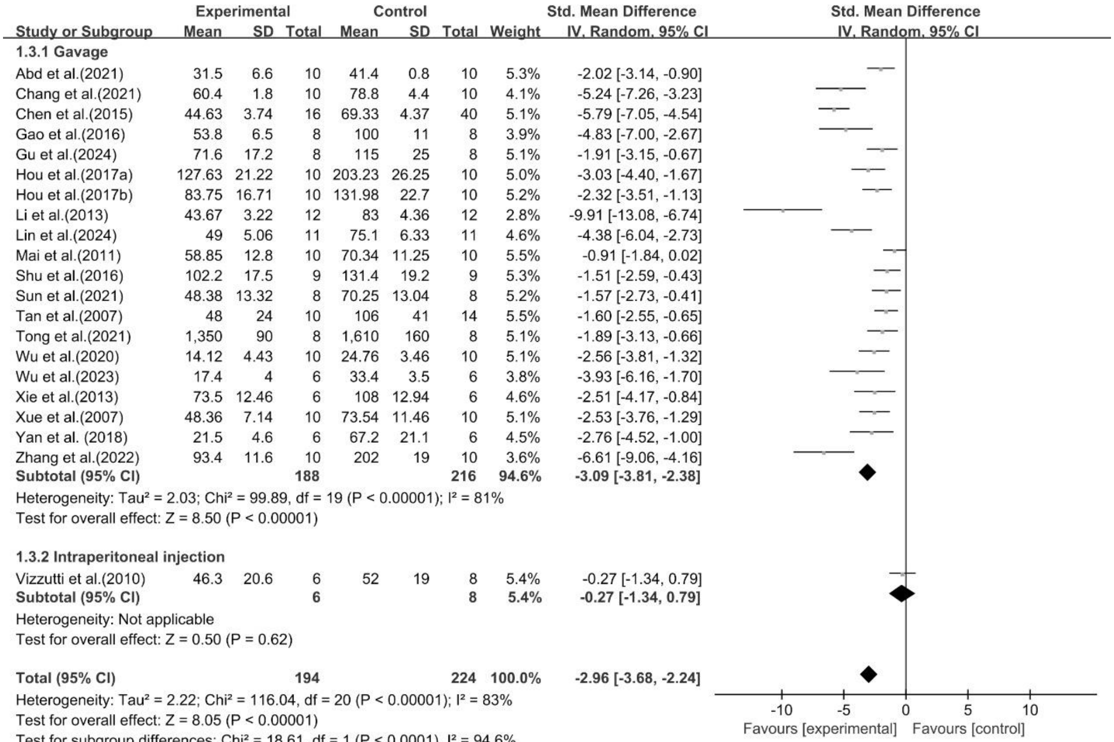

Supplementary Figure 23 Forest plot of NAS comparison for subgroup of different types of administration

Study or Subgroup	Experimental Mean	Experimental SD	Experimental Total	Control Mean	Control SD	Control Total	Weight	Std. Mean Difference IV, Random, 95% CI
**1.22.1 Gavage**								
Li et al.(2013)	1.8	0.84	12	2.67	0.52	12	25.8%	-1.20 [-2.08, -0.32]
Lin et al.(2024)	1.97	1	11	3.43	1.52	11	25.2%	-1.09 [-2.00, -0.18]
Wu et al.(2023)	2.32	0.49	6	6.08	0.75	6	5.8%	-5.48 [-8.38, -2.57]
Xue et al.(2007)	3.32	1.36	10	5.21	2.08	10	24.5%	-1.03 [-1.98, -0.08]
**Subtotal (95% CI)**			**39**			**39**	**81.3%**	**-1.48 [-2.44, -0.53]**
Heterogeneity: Tau² = 0.56; Chi² = 8.48, df = 3 (P = 0.04); I² = 65%								
Test for overall effect: Z = 3.05 (P = 0.002)								
**1.22.2 Intraperitoneal injection**								
Vizzutti et al.(2010)	0.48	0.62	6	1.44	0.5	8	18.7%	-1.62 [-2.90, -0.35]
**Subtotal (95% CI)**			**6**			**8**	**18.7%**	**-1.62 [-2.90, -0.35]**
Heterogeneity: Not applicable								
Test for overall effect: Z = 2.50 (P = 0.01)								
**Total (95% CI)**			**45**			**47**	**100.0%**	**-1.46 [-2.22, -0.70]**
Heterogeneity: Tau² = 0.38; Chi² = 8.76, df = 4 (P = 0.07); I² = 54%								
Test for overall effect: Z = 3.77 (P = 0.0002)								
Test for subgroup differences: Chi² = 0.03, df = 1 (P = 0.86), I² = 0%								

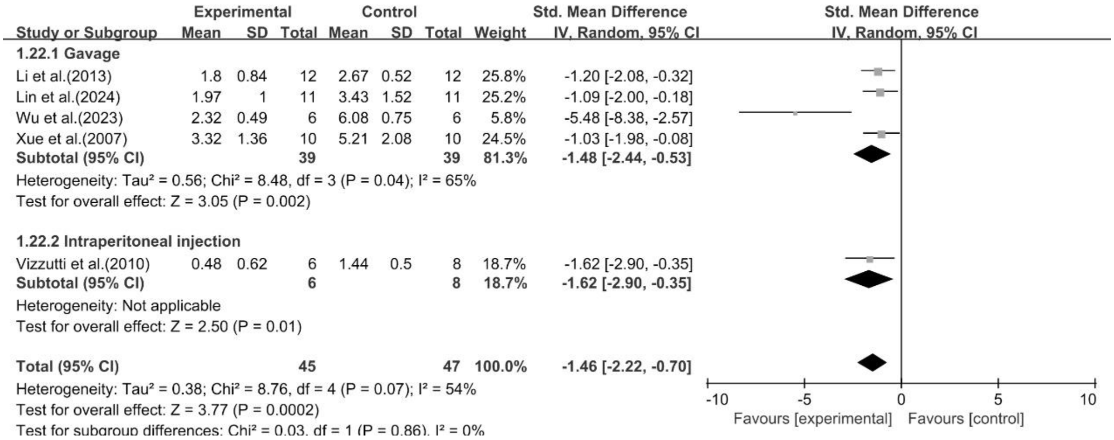

Supplementary Figure 24 Forest plot of ALT comparison for subgroup of different durations

Study or Subgroup	Experimental Mean	Experimental SD	Experimental Total	Control Mean	Control SD	Control Total	Weight	Std. Mean Difference IV, Random, 95% CI
**1.7.1 <8w**								
Abd et al.(2021)	31.5	6.6	10	41.4	0.8	10	5.3%	-2.02 [-3.14, -0.90]
Chen et al.(2015)	44.63	3.74	16	69.33	4.37	40	5.1%	-5.79 [-7.05, -4.54]
Gao et al.(2016)	53.8	6.5	8	100	11	8	3.9%	-4.83 [-7.00, -2.67]
Li et al.(2013)	43.67	3.22	12	83	4.36	12	2.8%	-9.91 [-13.08, -6.74]
Lin et al.(2024)	49	5.06	11	75.1	6.33	11	4.6%	-4.38 [-6.04, -2.73]
Shu et al.(2016)	102.2	17.5	9	131.4	19.2	9	5.3%	-1.51 [-2.59, -0.43]
Xie et al.(2013)	73.5	12.46	6	108	12.94	6	4.6%	-2.51 [-4.17, -0.84]
Xue et al.(2007)	48.36	7.14	10	73.54	11.46	10	5.1%	-2.53 [-3.76, -1.29]
Yan et al. (2018)	21.5	4.6	6	67.2	21.1	6	4.5%	-2.76 [-4.52, -1.00]
Zhang et al.(2022)	93.4	11.6	10	202	19	10	3.6%	-6.61 [-9.06, -4.16]
**Subtotal (95% CI)**			**98**			**122**	**44.8%**	**-4.01 [-5.29, -2.72]**
Heterogeneity: Tau² = 3.47; Chi² = 60.73, df = 9 (P < 0.00001); I² = 85%								
Test for overall effect: Z = 6.12 (P < 0.00001)								
**1.7.2 ≥8W**								
Chang et al.(2021)	60.4	1.8	10	78.8	4.4	10	4.1%	-5.24 [-7.26, -3.23]
Gu et al.(2024)	71.6	17.2	8	115	25	8	5.1%	-1.91 [-3.15, -0.67]
Hou et al.(2017a)	127.63	21.22	10	203.23	26.25	10	5.0%	-3.03 [-4.40, -1.67]
Hou et al.(2017b)	83.75	16.71	10	131.98	22.7	10	5.2%	-2.32 [-3.51, -1.13]
Mai et al.(2011)	58.85	12.8	10	70.34	11.25	10	5.5%	-0.91 [-1.84, 0.02]
Sun et al.(2021)	48.38	13.32	8	70.25	13.04	8	5.2%	-1.57 [-2.73, -0.41]
Tan et al.(2007)	48	24	10	106	41	14	5.5%	-1.60 [-2.55, -0.65]
Tong et al.(2021)	1,350	90	8	1,610	160	8	5.1%	-1.89 [-3.13, -0.66]
Vizzutti et al.(2010)	46.3	20.6	6	52	19	8	5.4%	-0.27 [-1.34, 0.79]
Wu et al.(2020)	14.12	4.43	10	24.76	3.46	10	5.1%	-2.56 [-3.81, -1.32]
Wu et al.(2023)	17.4	4	6	33.4	3.5	6	3.8%	-3.93 [-6.16, -1.70]
**Subtotal (95% CI)**			**96**			**102**	**55.2%**	**-2.08 [-2.75, -1.41]**
Heterogeneity: Tau² = 0.85; Chi² = 31.80, df = 10 (P = 0.0004); I² = 69%								
Test for overall effect: Z = 6.08 (P < 0.00001)								
**Total (95% CI)**			**194**			**224**	**100.0%**	**-2.96 [-3.68, -2.24]**
Heterogeneity: Tau² = 2.22; Chi² = 116.04, df = 20 (P < 0.00001); I² = 83%								
Test for overall effect: Z = 8.05 (P < 0.00001)								
Test for subgroup differences: Chi² = 6.80, df = 1 (P = 0.009), I² = 85.3%								

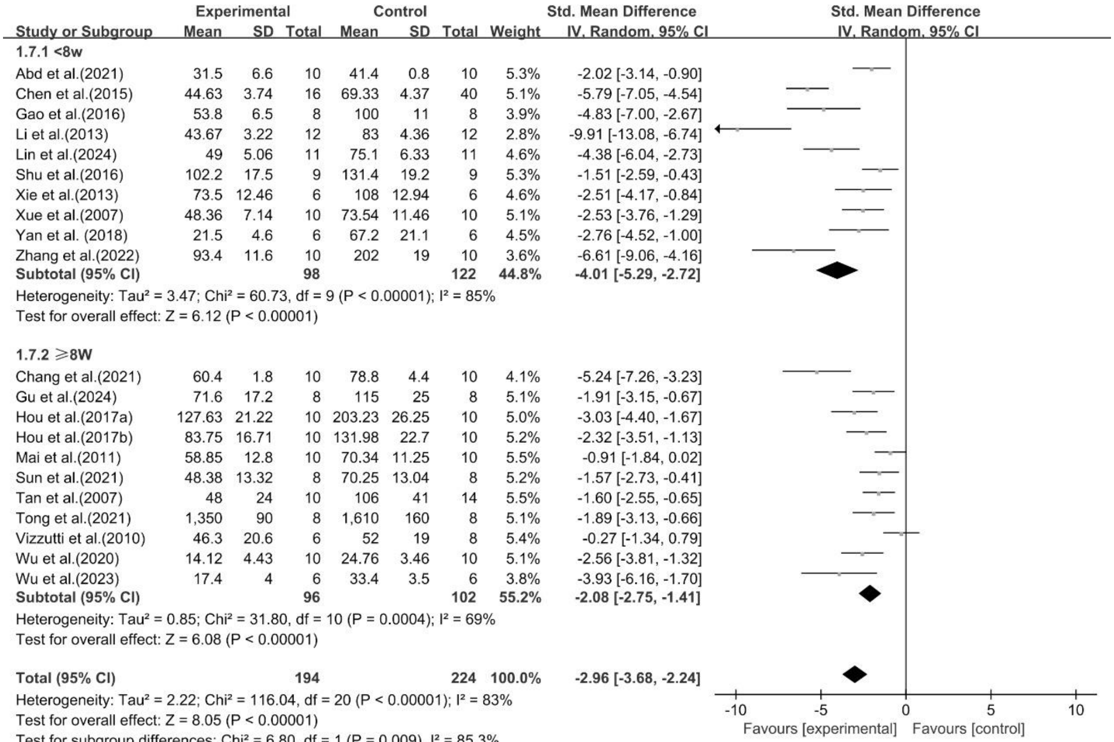

Supplementary Figure 25 Forest plot of AST comparison for subgroup of different durations

Study or Subgroup	Experimental Mean	Experimental SD	Experimental Total	Control Mean	Control SD	Control Total	Weight	Std. Mean Difference IV, Random, 95% CI
**1.6.1 <8w**								
Abd et al.(2021)	20.2	3.6	10	50.3	1.9	10	3.2%	-10.02 [-13.59, -6.44]
Chen et al.(2015)	161.72	8.44	16	259.98	13.77	40	5.6%	-7.74 [-9.33, -6.14]
Gao et al.(2016)	144	9	8	227	25	8	5.1%	-4.18 [-6.11, -2.24]
Li et al.(2013)	82.5	7.42	12	122.33	3.51	12	4.8%	-6.63 [-8.83, -4.42]
Lin et al.(2024)	162.7	8.88	11	200.5	8.8	11	5.6%	-4.11 [-5.69, -2.53]
Shu et al.(2016)	61.2	15.7	9	81.7	11.2	9	6.2%	-1.43 [-2.50, -0.37]
Xie et al.(2013)	193.83	34.77	6	305.83	64.27	6	5.7%	-2.00 [-3.50, -0.51]
Zhang et al.(2022)	107	25	10	235	20	10	5.0%	-5.42 [-7.48, -3.35]
**Subtotal (95% CI)**			**82**			**106**	**41.3%**	**-4.98 [-6.90, -3.06]**
Heterogeneity: Tau² = 6.65; Chi² = 68.44, df = 7 (P < 0.00001); I² = 90%								
Test for overall effect: Z = 5.08 (P < 0.00001)								
**1.6.2 ≥8W**								
Chang et al.(2021)	139	8	10	182	9	10	5.2%	-4.84 [-6.73, -2.95]
Gu et al.(2024)	103	34	8	182	43	8	6.0%	-1.93 [-3.17, -0.68]
Hou et al.(2017a)	83.75	16.71	10	131.98	22.7	10	6.1%	-2.32 [-3.51, -1.13]
Hou et al.(2017b)	127.63	21.22	10	203.23	26.25	10	5.9%	-3.03 [-4.40, -1.67]
Mai et al.(2011)	66.86	8.77	10	78.68	15.06	10	6.4%	-0.92 [-1.85, 0.01]
Sun et al.(2021)	140.38	16.24	8	189.63	20.5	8	5.8%	-2.52 [-3.92, -1.11]
Tan et al.(2007)	146	27	10	254	81	14	6.3%	-1.61 [-2.57, -0.66]
Tong et al.(2021)	976	64	8	1,170	120	8	6.0%	-1.91 [-3.15, -0.67]
Wu et al.(2020)	36.66	3.66	10	51.46	4.47	10	5.7%	-3.47 [-4.96, -1.98]
Wu et al.(2023)	22.7	2.1	6	29.6	2.1	6	5.2%	-3.03 [-4.90, -1.17]
**Subtotal (95% CI)**			**90**			**94**	**58.7%**	**-2.38 [-3.02, -1.74]**
Heterogeneity: Tau² = 0.60; Chi² = 21.80, df = 9 (P = 0.010); I² = 59%								
Test for overall effect: Z = 7.32 (P < 0.00001)								
**Total (95% CI)**			**172**			**200**	**100.0%**	**-3.46 [-4.34, -2.58]**
Heterogeneity: Tau² = 2.94; Chi² = 113.69, df = 17 (P < 0.00001); I² = 85%								
Test for overall effect: Z = 7.70 (P < 0.00001)								
Test for subgroup differences: Chi² = 6.33, df = 1 (P = 0.01), I² = 84.2%								

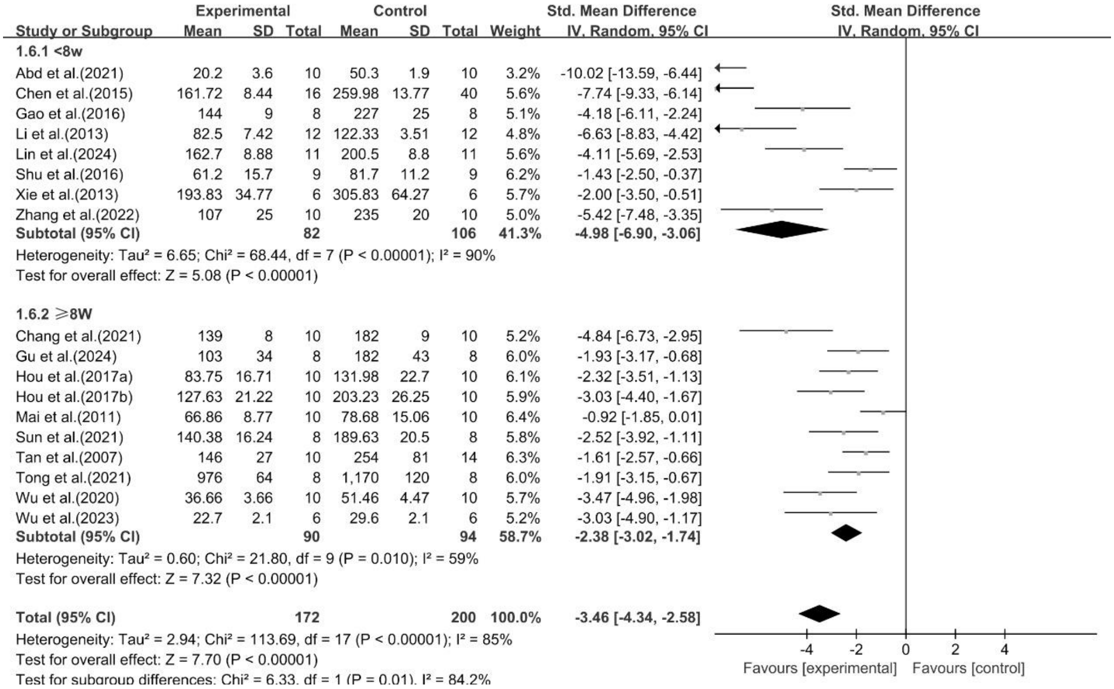

Supplementary Figure 26 Forest plot of TC comparison for subgroup of different durations

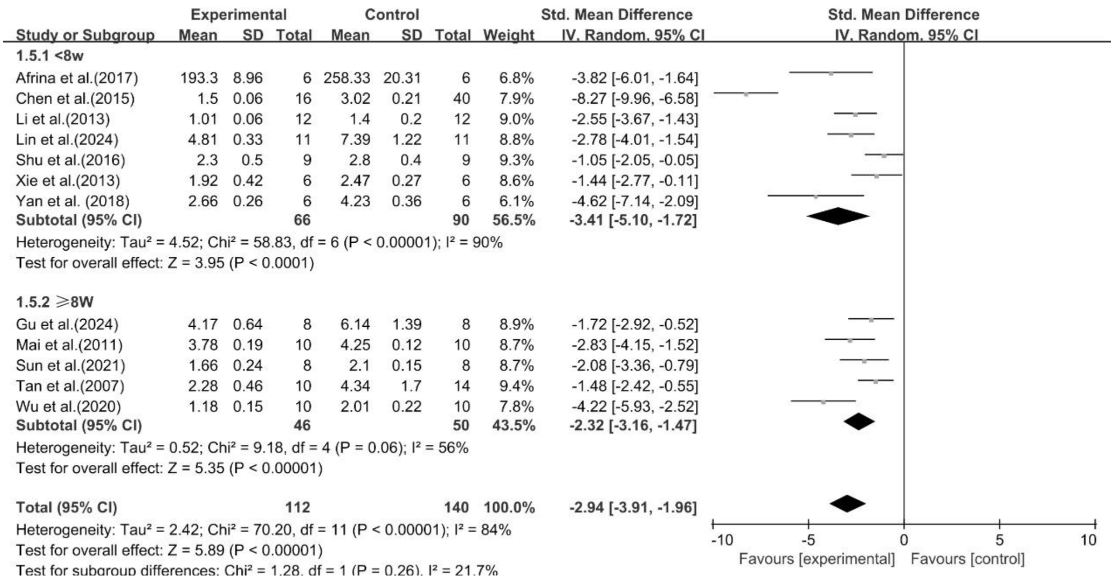

Supplementary Figure 27 Forest plot of TG comparison for subgroup of different durations

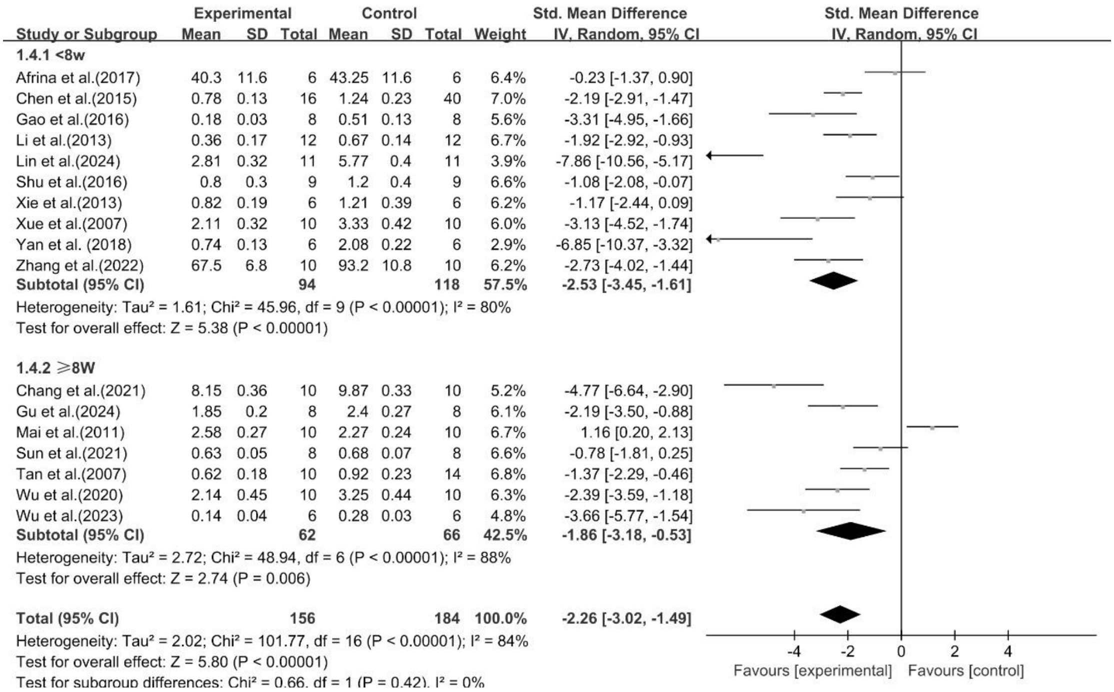

Supplementary Figure 28 Forest plot of HDL comparison for subgroup of different durations

	Experimental			Control				Std. Mean Difference
Study or Subgroup	Mean	SD	Total	Mean	SD	Total	Weight	IV, Random, 95% CI
**1.3.1 <8w**								
Lin et al.(2024)	1.5	0.26	11	1.04	0.15	11	15.2%	2.08 [1.01, 3.16]
Xie et al.(2013)	0.95	0.08	6	0.83	0.13	6	13.9%	1.03 [-0.21, 2.26]
Xue et al.(2007)	1.23	0.21	10	0.89	0.12	10	15.0%	1.90 [0.81, 3.00]
**Subtotal (95% CI)**			**27**			**27**	**44.1%**	**1.73 [1.07, 2.38]**
Heterogeneity: Tau² = 0.00; Chi² = 1.76, df = 2 (P = 0.42); I² = 0%								
Test for overall effect: Z = 5.18 (P < 0.00001)								
**1.3.2 ≥8W**								
Gu et al.(2024)	1.53	0.24	8	1.14	0.18	8	14.2%	1.74 [0.54, 2.94]
Sun et al.(2021)	0.92	0.1	8	0.81	0.11	8	15.4%	0.99 [-0.07, 2.05]
Tan et al.(2007)	0.28	0.16	10	0.29	0.17	14	17.5%	-0.06 [-0.87, 0.75]
Wu et al.(2023)	1.06	0.09	6	0.81	0.04	6	8.9%	3.31 [1.34, 5.29]
**Subtotal (95% CI)**			**32**			**36**	**55.9%**	**1.29 [0.07, 2.51]**
Heterogeneity: Tau² = 1.14; Chi² = 12.96, df = 3 (P = 0.005); I² = 77%								
Test for overall effect: Z = 2.07 (P = 0.04)								
**Total (95% CI)**			**59**			**63**	**100.0%**	**1.43 [0.66, 2.19]**
Heterogeneity: Tau² = 0.70; Chi² = 18.81, df = 6 (P = 0.005); I² = 68%								
Test for overall effect: Z = 3.66 (P = 0.0003)								
Test for subgroup differences: Chi² = 0.39, df = 1 (P = 0.53), I² = 0%								

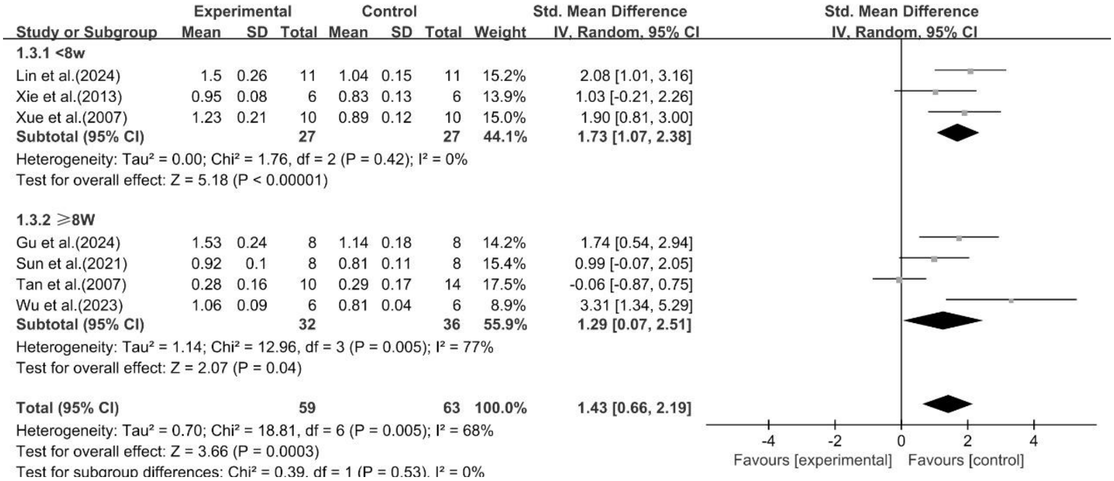

Supplementary Figure 29 Forest plot of LDL comparison for subgroup of different durations

	Experimental			Control				Std. Mean Difference
Study or Subgroup	Mean	SD	Total	Mean	SD	Total	Weight	IV, Random, 95% CI
**1.2.1 <8w**								
Lin et al.(2024)	0.65	0.05	11	0.98	0.11	11	18.7%	-3.72 [-5.19, -2.24]
Xie et al.(2013)	0.4	0.07	6	0.7	0.13	6	16.4%	-2.65 [-4.37, -0.93]
**Subtotal (95% CI)**			**17**			**17**	**35.1%**	**-3.27 [-4.38, -2.15]**
Heterogeneity: Tau² = 0.00; Chi² = 0.85, df = 1 (P = 0.36); I² = 0%								
Test for overall effect: Z = 5.72 (P < 0.00001)								
**1.2.2 ≥8W**								
Gu et al.(2024)	1.88	0.25	8	2.43	0.57	8	22.6%	-1.18 [-2.27, -0.09]
Tan et al.(2007)	0.29	0.3	10	0.73	0.52	14	24.9%	-0.96 [-1.82, -0.09]
Wu et al.(2023)	0.39	0.04	6	0.55	0.08	6	17.4%	-2.34 [-3.94, -0.73]
**Subtotal (95% CI)**			**24**			**28**	**64.9%**	**-1.26 [-1.92, -0.59]**
Heterogeneity: Tau² = 0.03; Chi² = 2.21, df = 2 (P = 0.33); I² = 9%								
Test for overall effect: Z = 3.71 (P = 0.0002)								
**Total (95% CI)**			**41**			**45**	**100.0%**	**-2.04 [-3.07, -1.01]**
Heterogeneity: Tau² = 0.91; Chi² = 12.68, df = 4 (P = 0.01); I² = 68%								
Test for overall effect: Z = 3.89 (P < 0.0001)								
Test for subgroup differences: Chi² = 9.16, df = 1 (P = 0.002), I² = 89.1%								

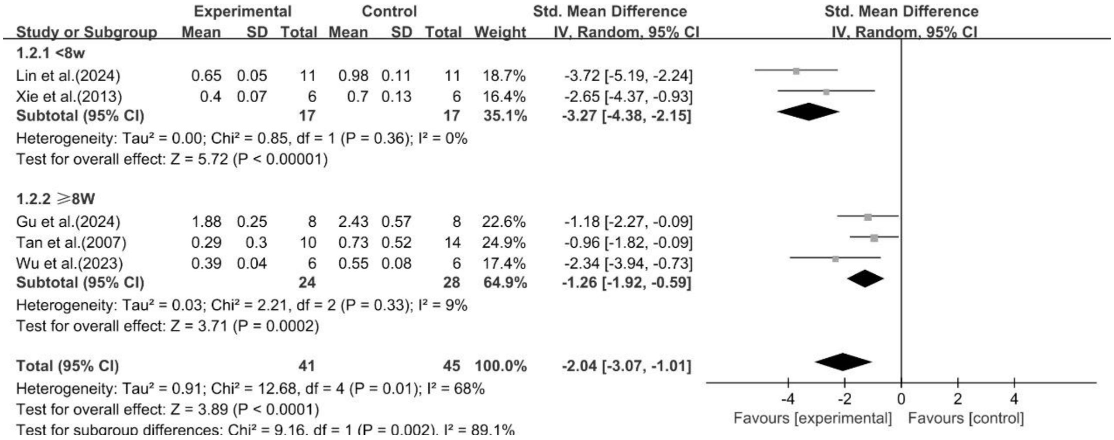

Supplementary Figure 30 Forest plot of NAS comparison for subgroup of different durations

	Experimental			Control				Std. Mean Difference
Study or Subgroup	Mean	SD	Total	Mean	SD	Total	Weight	IV, Random, 95% CI
**1.1.1 <8w**								
Li et al.(2013)	1.8	0.84	12	2.67	0.52	12	25.7%	-1.20 [-2.08, -0.32]
Lin et al.(2024)	1.97	1	11	3.43	1.52	11	25.2%	-1.09 [-2.00, -0.18]
Xue et al.(2007)	3.32	1.36	10	5.21	2.08	10	24.5%	-1.03 [-1.98, -0.08]
**Subtotal (95% CI)**			**33**			**33**	**75.4%**	**-1.11 [-1.64, -0.59]**
Heterogeneity: Tau² = 0.00; Chi² = 0.07, df = 2 (P = 0.97); I² = 0%								
Test for overall effect: Z = 4.14 (P < 0.0001)								
**1.1.2 ≥8W**								
Vizzutti et al.(2010)	0.48	0.62	6	1.44	0.5	8	19.0%	-1.62 [-2.90, -0.35]
Wu et al.(2023)	2.12	0.49	6	6.18	0.75	6	5.5%	-5.92 [-9.02, -2.81]
**Subtotal (95% CI)**			**12**			**14**	**24.6%**	**-3.53 [-7.71, 0.65]**
Heterogeneity: Tau² = 7.74; Chi² = 6.29, df = 1 (P = 0.01); I² = 84%								
Test for overall effect: Z = 1.65 (P = 0.10)								
**Total (95% CI)**			**45**			**47**	**100.0%**	**-1.47 [-2.26, -0.68]**
Heterogeneity: Tau² = 0.43; Chi² = 9.32, df = 4 (P = 0.05); I² = 57%								
Test for overall effect: Z = 3.66 (P = 0.0003)								
Test for subgroup differences: Chi² = 1.26, df = 1 (P = 0.26), I² = 20.9%								

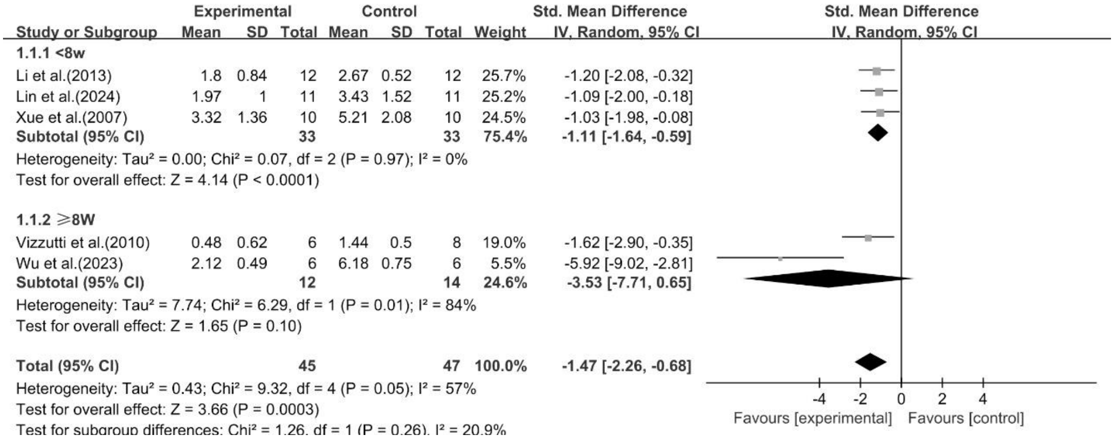

Supplement: Supplementary file 2 [file DataSheet1.pdf]
